# Efficacy and safety of gut microbiota-based therapies in autoimmune and rheumatic diseases: a systematic review and meta-analysis of 80 randomized controlled trials

**DOI:** 10.1186/s12916-024-03303-4

**Published:** 2024-03-13

**Authors:** Liuting Zeng, Kailin Yang, Qi He, Xiaofei Zhu, Zhiyong Long, Yang Wu, Junpeng Chen, Yuwei Li, Jinsong Zeng, Ge Cui, Wang Xiang, Wensa Hao, Lingyun Sun

**Affiliations:** 1 Department of Rheumatology and Immunology, Nanjing Drum Tower Hospital, Chinese Academy of Medical Sciences and Peking Union Medical College, Graduate School of Peking Union Medical College, Nanjing, China; 2 Hunan University of Chinese Medicine, Changsha, China; 3 People’s Hospital of Ningxiang City, Ningxiang, China; 4 Fudan University, Shanghai, China; 5 Department of Rehabilitation Medicine, Guangzhou Panyu Central Hospital, Guangzhou, China; 6 Department of Rheumatology, National Clinical Research Center for Dermatologic and Immunologic Diseases, State Key Laboratory of Complex Severe and Rare Diseases, Peking Union Medical College Hospital, Chinese Academy of Medical Science and Peking Union Medical College, Beijing, China; 7 University of Louisville, Louisville, USA; 8 Hunan University of Science and Technology, Xiangtan, China; 9 Department of Epidemiology and Statistics, School of Public Health, Peking Union Medical College Hospital, Chinese Academy of Medical Sciences & Peking Union Medical College, Beijing, China; 10 Department of Rheumatology, The First People’s Hospital Changde City, Changde, China; 11 Institute of Materia Medica, Chinese Academy of Medical Sciences and Peking Union Medical College, Beijing, China; 12 Department of Rheumatology and Immunology, The First Affiliated Hospital of Anhui Medical University, Hefei, China

**Keywords:** Autoimmune disease, Probiotics, Gut microbiota-based therapies, Systematic review, Meta-analysis

## Abstract

**Background:**

Previous randomized controlled trials (RCTs) suggested that gut microbiota-based therapies may be effective in treating autoimmune diseases, but a systematic summary is lacking.

**Methods:**

Pubmed, EMbase, Sinomed, and other databases were searched for RCTs related to the treatment of autoimmune diseases with probiotics from inception to June 2022. RevMan 5.4 software was used for meta-analysis after 2 investigators independently screened literature, extracted data, and assessed the risk of bias of included studies.

**Results:**

A total of 80 RCTs and 14 types of autoimmune disease [celiac sprue, SLE, and lupus nephritis (LN), RA, juvenile idiopathic arthritis (JIA), spondyloarthritis, psoriasis, fibromyalgia syndrome, MS, systemic sclerosis, type 1 diabetes mellitus (T1DM), oral lichen planus (OLP), Crohn’s disease, ulcerative colitis] were included. The results showed that gut microbiota-based therapies may improve the symptoms and/or inflammatory factor of celiac sprue, SLE and LN, JIA, psoriasis, PSS, MS, systemic sclerosis, Crohn’s disease, and ulcerative colitis. However, gut microbiota-based therapies may not improve the symptoms and/or inflammatory factor of spondyloarthritis and RA. Gut microbiota-based therapies may relieve the pain of fibromyalgia syndrome, but the effect on fibromyalgia impact questionnaire score is not significant. Gut microbiota-based therapies may improve HbA1c in T1DM, but its effect on total insulin requirement does not seem to be significant. These RCTs showed that probiotics did not increase the incidence of adverse events.

**Conclusions:**

Gut microbiota-based therapies may improve several autoimmune diseases (celiac sprue, SLE and LN, JIA, psoriasis, fibromyalgia syndrome, PSS, MS, T1DM, Crohn’s disease, and ulcerative colitis).

**Supplementary Information:**

The online version contains supplementary material available at 10.1186/s12916-024-03303-4.

## Background

Autoimmune diseases are chronic inflammatory diseases caused by the breakdown of autoimmune tolerance; T cells and antibodies react with their own cells and tissue antigens, resulting in loss or limitation of tissue function. The mechanism by which autoimmune tolerance is broken has not yet been clarified [1, 2]. Autoimmune diseases have a broad spectrum, and nearly 100 diseases have been found to have an autoimmune basis, including rheumatoid arthritis (RA), systemic lupus erythematosus (SLE), and multiple sclerosis (MS). There are at least 80 other diseases that may be associated with autoimmunity [3–5]. Epidemiology shows that the global incidence of autoimmune diseases is about 0.09%. In most autoimmune diseases, the incidence of women is significantly higher than that of men, and the overall incidence is increasing [6–8]. Evidence shows that its occurrence is closely related to genetic, environmental, intestinal flora, and other factors [9–11]. The “fecal transplantation” technology has been widely used in the treatment of autoimmune diseases such as ulcerative colitis because it can change the composition and diversity of intestinal flora, but this technique is still limited because there are few studies on the relative changes of donor and recipient microbiota after transplantation [12–14]. The current drugs for the treatment of autoimmune diseases mainly include glucocorticoids and immunosuppressants such as disease-modifying antirheumatic drugs (DMARDs). DMARDs mainly include conventional synthetic DMARDs such as methotrexate and leflunomide and biological DMARDs such as TNF-α inhibitors, IL-6 and IL-6 receptor inhibitors, anti-CD20 antibodies, and targeted synthetic DMARDs [15]. The main treatment drugs for SLE are rituximab, belimumab (an anti-B cell activating factor monoclonal antibody), etc. [16, 17]. Common therapeutic drugs for MS include IFN-β preparations and glatiramer acetate [18]. Although traditional glucocorticoids and immunosuppressants can inhibit the disease and improve the survival rate of patients, long-term use will cause a series of adverse consequences, and there are more serious adverse reactions [19, 20]. Therefore, it is necessary to achieve breakthroughs in the treatment of autoimmune diseases in drug molecular pathways and targets, which are both a challenge and an opportunity.

Probiotics are a general term for a class of active microorganisms that can colonize the host intestine and have beneficial effects on the body. By interacting with host cells, they affect the composition and structural integrity of the intestinal flora, thereby affecting their metabolism and immunity [19]. WHO defines probiotics as “live microorganisms that, when administered in sufficient amounts, confer a health benefit to the host” [19]. Probiotics have been used to treat a variety of gastrointestinal diseases. The most commonly used probiotics are *Lactobacillus* (such as *Lactobacillus casei*, *Lactobacillus rhamnosus*, *Lactobacillus plantarum*, *Lactobacillus helveticus*), *Bifidobacterium* (such as *Bifidobacterium breve*, *Bifidobacterium longum*, *Bifidobacterium infantis*), and *Saccharomyces boulardii* [21, 22]. However, there is no consensus on the role of various probiotics, and there is still controversy about the safety of probiotics. For example, lactic acid bacteria have long been used in food processing and have proven their safety [23]. At present, a large number of clinical trials, animal models, and in vitro studies have found that probiotics can effectively treat autoimmune diseases through a variety of immune pathways [24–27]. Due to the complexity of the pathogenesis of autoimmune diseases, as well as the individual differences of probiotics, different types and doses of treatment and even the different growth status of probiotics, the immune regulation ability of probiotics is different. The evidence of clinical use of probiotics in the treatment of autoimmune diseases is relatively confusing, and it cannot give better guidance to clinical practice. Therefore, this study hopes to conduct a comprehensive summary of randomized controlled trials (RCTs) of probiotics in the treatment of autoimmune diseases, so as to provide solid evidence for clinical practice, and to provide more references for the design of future RCTs.

## Methods

### Protocol

This systematic review and meta-analysis was performed strictly according to protocols registered in the PROSPERO (CRD42023466683) and PRISMA guidelines (see Additional file 1) [28].

### Literature sources

China National Knowledge Infrastructure (CNKI), Medline Complete, Pubmed, Web of Science, Sinomed, VIP Database, Wanfang Database, and EMbase were searched for literature on gut microbiota-based therapies for the treatment of autoimmune diseases. The retrieval time is from the establishment of the database to Oct 1st, 2023. We also searched ClinicalTrials.gov and Cochrane Library. The search strategy was shown in the supplementary material table (see Additional file 2).

### Search criteria

#### Participants

Patients were diagnosed with any autoimmune disease by accepted criteria. Autoimmune diseases include but are not limited to celiac sprue, SLE and lupus nephritis (LN), RA, juvenile idiopathic arthritis (JIA), spondyloarthritis, psoriasis, fibromyalgia syndrome, MS, systemic sclerosis, type 1 diabetes (T1DM), oral lichen planus (OLP), Crohn’s disease, and ulcerative colitis.

#### Intervention methods

The experimental group used probiotic preparations, which could be used alone or in combination, while the control group used the therapy without probiotics. The type and content of probiotics, the duration of intervention, and the route of administration are not limited.

#### Outcomes

Outcomes are the efficacy indicators of the disease (such as SLEDAI, DAS28, PASI score), inflammatory factor indicators and adverse events.

#### Study design

The design of the study was an RCT, and there were no restrictions on the method of random sequence generation, the year of publication, and the language of the literature.

#### Exclusion criteria

(1) The type of target literature does not match, such as review, animal experiments, data mining, or non-RCT; (2) The research disease or medication method is inconsistent; (3) The evaluation criteria do not meet the inclusion requirements; (4) The control group adopted the intervention measures containing probiotics.

### Search screening methods

(1) Preliminary screening of literatures: The literatures screened by the search strategy were assigned to two researchers, who read the literature titles and abstracts respectively, and excluded non-clinical studies that did not belong to the treatment of autoimmune diseases with probiotics. (2) Then carry out literature rescreening: further preliminary screening of the full text of the literature refers to the inclusion and exclusion criteria to determine the final included RCTs. In case of disagreement between two researchers in the selection of literature, the decision shall be discussed with all researchers.

### Quality assessments and data extraction

The included RCTs were quantitatively assessed according to the Cochrane Risk of Bias Tools. For the possible sources of bias risk arising from improper experimental methods or the limitations of the sample itself in the research process, three evaluations are given: high risk, inability to judge, and low risk. Revman 5.4 software was used to generate percent risk of bias graphs and summary risk of bias graphs [29].

The basic information and clinical index data in the text and chart of RCT were manually entered. It mainly includes the basic information of the literature: title, author, publication time, basic information of research subjects (sample size and age of patients), treatment method (type of probiotics or drug name, dose, course of treatment), outcome indicators, and adverse reactions. If any data was missing, it would be obtained by extrapolation or by trying to communicate with the original author [30].

The above operations were performed independently by two researchers. In case of disagreement between two researchers in the selection of literature, the decision shall be discussed with all researchers.

### Statistical analysis

Data were processed using RevMan5.4 software [31]. Data on dichotomous variables were studied using relative risk (RR). Weighted mean differences (WMD) were used to study continuous variables with uniform measurement units; standardized mean differences (SMD) were used to study continuous variables with non-uniform measurement units. The intergroup heterogeneity of the selected studies was tested and analyzed. When the inter-study heterogeneity was small (*P*>0.05, *I*^2^≤50%), the model was robust and the heterogeneity was small; at this time, the data were combined using a fixed-effects model. Heterogeneity was present if the between-study heterogeneity was large (*P*≤0.05, *I*^2^>50%); at this time, the data were combined and analyzed using a random-effects model, with 95% confidence intervals (CI) [31]. STATA 15 was used to detect publication bias in outcomes of RCTs> 5 by Egger’s method (for continuous variables) and Harbord’s method (for dichotomous variables) [32]. *P*>0.1 was considered to have no publication bias.

## Results

### Search results

**Fig. 1:**
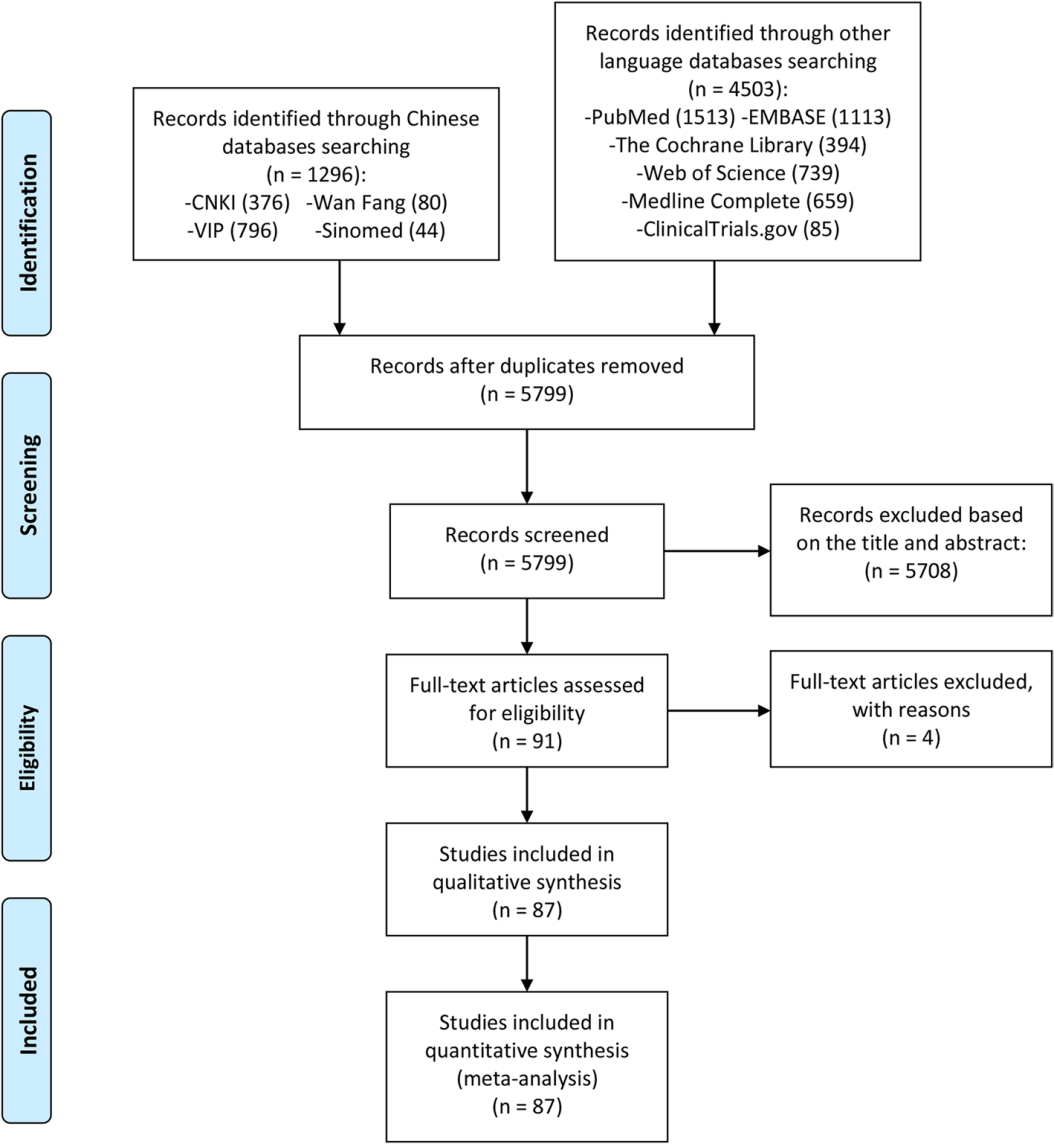
Flow diagram

A total of 5799 preliminary related literatures were detected in this study, and a total of 5708 literatures that did not conform to the research type and content were excluded. After the primary screening, 91 records were obtained. According to the search criteria and the completeness of the literature information, 4 records were excluded from the second screening after reading the full text [33–36], and 87 records [37–122] were finally included in the full text. The literature screening process and results are shown in Fig. 1.

### Characteristics of included records

Three trials were recorded as Primec et al. [37–39] for they came from the same RCT. The other two trials were recorded as Oscarsson et al. [40, 41], Roman et al. [72, 73], and Kragelund [88, 89], and the other three trials were recorded as Alipour et al. [51–53] for the same reason. As a result, a total of 80 RCT studies were examined. Some RCTs contain 2 experimental groups and are therefore divided into a and b. For example, Ma et al. [95] was divided into Ma et al. 2020a and Ma et al. 2020b during meta-analysis, and its control group was divided in half to match the two experimental groups. The included RCTs involved 14 autoimmune diseases (celiac sprue, Crohn’s disease, fibromyalgia syndrome, JIA, MS, OLP, psoriasis, primary Sjögren’s syndrome (PSS), RA, SLE and LN, spondyloarthritis, systemic sclerosis, T1DM, ulcerative colitis) and were from 27 different countries and regions [Slovenia, Sweden, Italy, China, Canada, Argentina, Australian, Spain, Iran, the USA, New Zealand, Finland, Brazil, India, the UK, Ireland, Spanish, Egypt, Singapore, Mexico, Taiwan (China), Poland, Denmark, Turkey, Germany, Japan, México]. The details of study characteristics are presented in a table (see Additional file 3).

### Risk of bias assessments

**Fig. 2:**
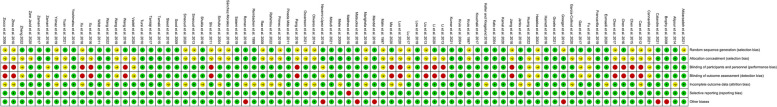
Risk of bias summary

**Fig. 3:**
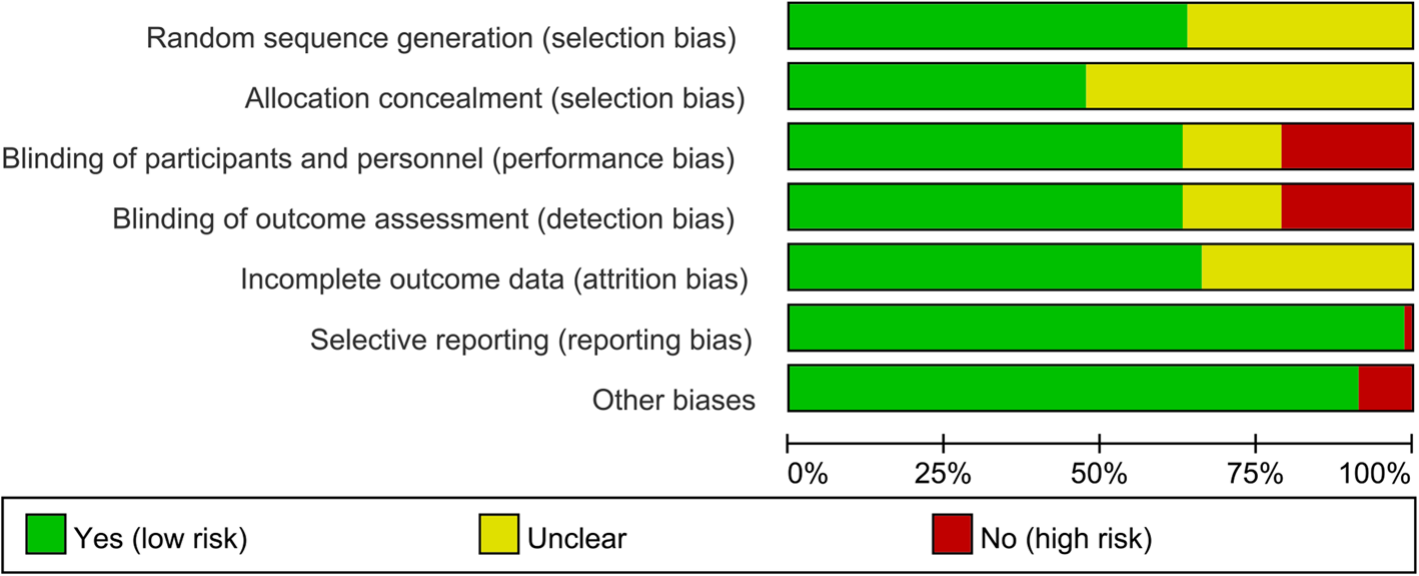
Risk of bias graph

The summary and graph of risk of bias are shown in Figs. 2 and 3.

#### Random sequence generation and allocation concealment

Twenty-nine RCTs failed to describe the random sequence generation methods; hence, they were rated as unclear risk of bias. Others were assessed as low risk of bias because they described the random sequence generation method. For allocation concealment, forty-two RCTs were assessed as unclear risk of bias for they did not clearly describe the allocation concealment methods, and other RCTs were rated as low risk of bias because they clearly describe the allocation concealment methods.

#### Blinding, incomplete outcome data and selective reporting

Seventeen RCTs were not blinded and their results contained subjective indicators, so they were rated as having a high risk of bias. Thirteen RCTs mentioned the use of blinding, but did not describe the implementation process in detail, and were therefore rated as unclear risk of bias. Other RCTs were rated as low risk of bias for they used blinding and described the implementation process, or the indicators were objective indicators. Twenty-seven RCTs had missing data and did not use appropriate statistical processing methods, so they were assessed as having unclear risk of bias. Matthes et al. [110] had outcomes that were not reported and were therefore assessed as having a high risk of bias in selective reporting. Other RCTs reported all results as described in the proposal or methodology and were therefore assessed as having a low risk of bias in selective reporting.

### Other potential bias

Seven RCTs were rated as high risk of bias: Brophy et al. [66] because the entire survey was conducted through the Internet and there was no contact with patients, so there may be bias; the remaining RCTs may be biased because some of the authors work in relevant companies. Other sources of bias were not observed in other RCTs and they were rated as low risk of bias.

### Gut microbiota-based therapies for celiac sprue

A total of 7 RCTs reported probiotic treatment of celiac sprue. Since the indicators of RCTs are not uniform, only a systematic review was conducted. Primec et al. [37–39] administered *Bifidobacterium breve* BR03 (DSM 16604) 1*10^9 CFU and *Bifidobacterium breve* B632 (DSM 24706) 1*10^9 CFU orally for 3 months and found negative correlations between *Verrucobacterium*, some unknown bacterial phyla, synergetic phyla, *Euryarchaeota*, and short-chain fatty acids (SCFAs) in the probiotic group. *Synergistetes* and *Euryarchaeota* may play a role in anti-inflammatory processes in the healthy human gut. They also found that *Verrucomicrobia*, *Parcubacteria*, and some unknown bacterial and archaeal phyla may be related to celiac sprue and have a strong correlation with TNF-α, and probiotics can reduce TNF-α levels. Oscarsson et al. [40, 41] used *L. plantarum* HEAL9 + *L. paracasei* 8700:2 (1*10^9 CFU) and found a decrease in transglutaminase autoantibodies (tTGA) in the probiotic group, which may be positively correlated with Dialister. They also found that the probiotic combination may modulate peripheral immune responses. Francavilla et al. [42] found that probiotics had significantly lower IBS-SSS and GSRS and significantly higher treatment success (*P*<0.05) compared with placebo, while *Lactobacillus*, *Staphylococcus*, and *Bifidobacterium* increased. In addition, they reported no adverse events. Smecuol et al. [43] found that the probiotic group had decreased IgA tTG and IgA DGP antibody concentrations and significantly increased serum macrophage inflammatory protein 1β (*P* < 0.05), and was relatively safe. Olivares et al. [39] found that peripheral CD3+ T lymphocytes decreased in the probiotic group (*P*<0.05). Compared with placebo, the number of *B. fragilis* and the content of sIgA in feces were decreased in the probiotic group (*P*<0.05).

While the above results suggest efficacy, Harnett et al. [44] showed no statistically significant changes in fecal microbiota counts or blood safety measures between the probiotic and placebo groups (*P* > 0.05). Smecuol et al. [45] found that *B. infantis* NLS-SS improved specific symptoms in only a subset of highly symptomatic treated patients, with no adverse effects in the two groups.

### Gut microbiota-based therapies for SLE and LN

#### SLEDAI

**Fig. 4:**
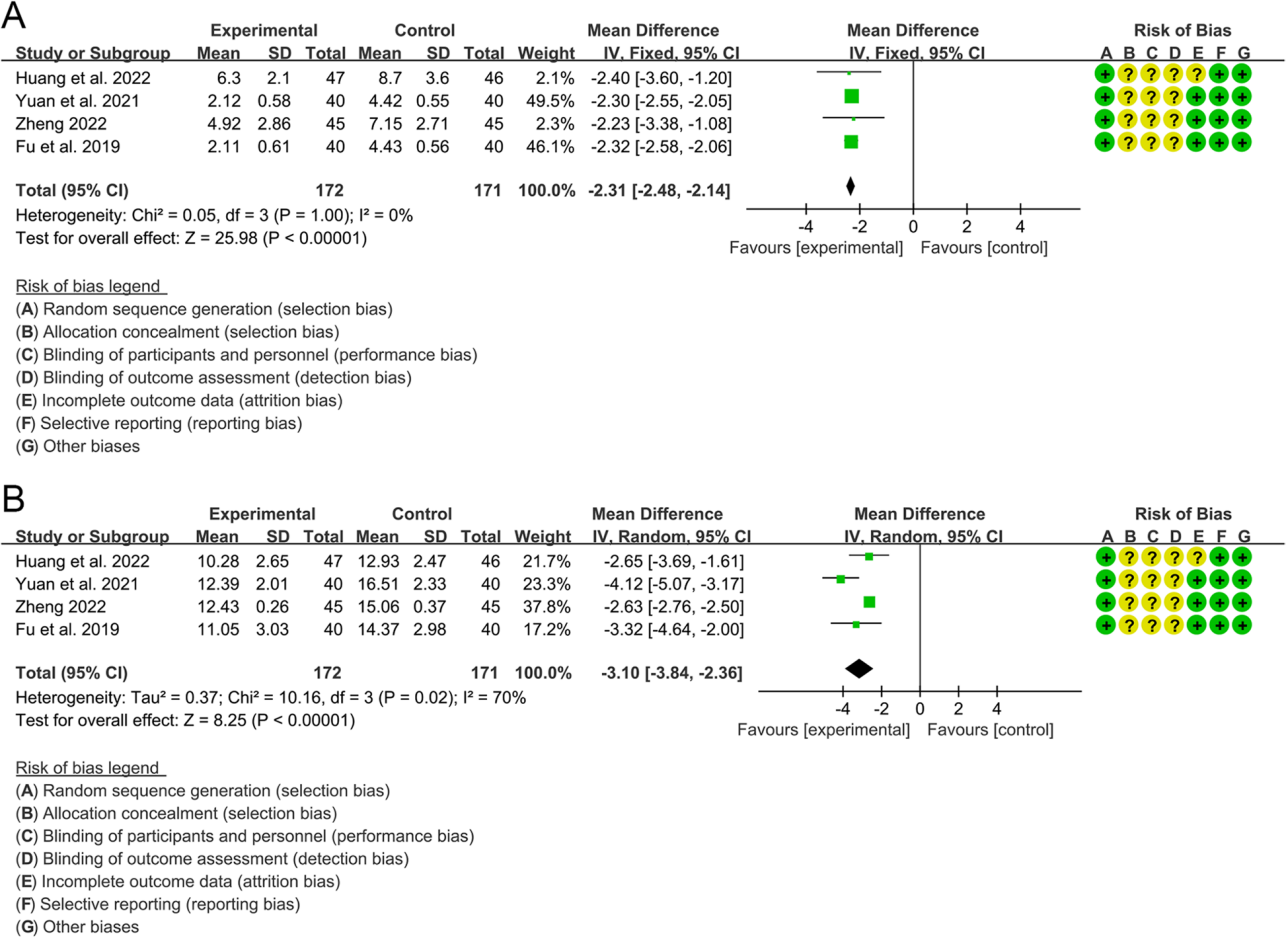
Outcomes of SLE and LN (**A** SLEDAI; **B** IgG level)

A total of 4 RCTs used SLEDAI as the outcome indicator. The heterogeneity between groups was low, and a fixed effect model was used (*I*^2^=0%, *P*=1.00). The results showed that compared with control group, SLEDAI in the experimental group was lower {WMD=−2.31, 95%CI [−2.48, −2.14], *P*<0.00001} (Fig. 4A).

#### Complement C3

A total of 2 RCTs used complement C3 as the outcome indicator. Zheng [48] found that compared with the control group, blood complement C3 levels were higher after treatment with gut microbiota-based therapy (*P*<0.05). Fu et al. [49] also found that the complement C3 level after gut microbiota-based therapy was higher than that in the control group (*P*<0.05). These suggest that gut microbiota-based therapy may increase complement C3 levels in SLE patients.

#### IgG level

A total of 4 RCTs used IgG as the outcome indicator. The heterogeneity between groups was high and a random effect model was used (*I*^2^=70%, *P*=0.02). The results showed that compared with control group, IgG level in the experimental group was lower {WMD=−3.10, 95%CI [−3.84, −2.86], *P*<0.00001} (Fig. 4B).

#### Adverse events of gut microbiota-based therapies for SLE and LN

Only Huang et al. [46] and Yuan et al. [47] reported adverse events. Huang et al. [46] showed that there was no significant difference in the incidence of abnormal liver function, infection (upper respiratory tract, lung, urinary tract), diarrhea, tachycardia, and other adverse drug reactions between the two groups of patients (31.91% in experiment group v.s. 34.78% in control group). Yuan et al. [47] showed that no associated adverse events were observed.

### Gut microbiota-based therapies for RA

#### DAS28

**Fig. 5:**
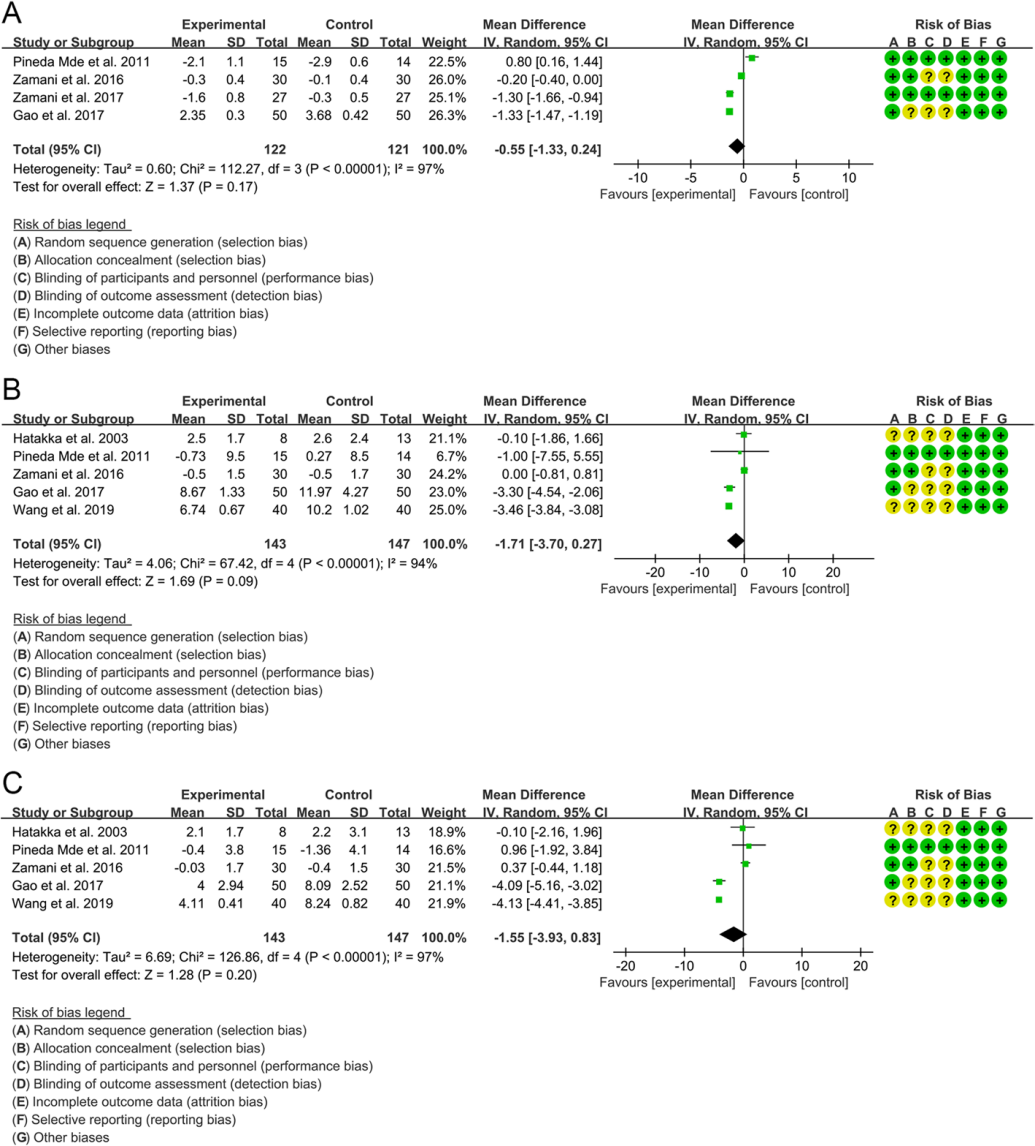
Outcomes of RA (**A** DAS28; **B** tender joint counts; **C** swollen joint counts)

Four RCTs reported analyzable data on DAS28. The heterogeneity test indicated that the heterogeneity among the included RCTs was high (*I*^2^=97%, *P*<0.00001). The results of meta-analysis showed that there was no significant difference in DAS28 between the probiotic group and the control group {WMD=−0.55, 95%CI [−1.33, 0.24], *P*=0.17} (Fig. 5A).

#### Tender joint counts and swollen joint counts

1. Tender joint count: Five RCTs reported analyzable data on tender joint counts. The heterogeneity test indicated that the heterogeneity among the included RCTs was high (*I*^2^=94%, *P*<0.00001). The results of meta-analysis showed that there was no significant difference in tender joint counts between the probiotic group and the control group {WMD=−1.71, 95%CI −3.70, 0.27], *P*=0.09} (Fig. 5B).
2. Swollen joint count: Five RCTs reported analyzable data on tender joint counts. The heterogeneity test indicated that the heterogeneity among the included RCTs was high (*I*^2^=97%, *P*<0.00001). The results of meta-analysis showed that there was no significant difference in swollen joint count between the probiotic group and the control group {WMD=−1.55, 95%CI [−3.93, 0.83], *P*=0.20} (Fig. 5C).

#### Adverse events of gut microbiota-based therapies for RA

Five RCTs reported adverse events. Mandel et al. [55], Alipour et al. [51–53], and Pineda Mde et al. [56] did not report any adverse events. Vadell et al. [59] observed 13 cases of gastrointestinal adverse events in the intervention group and 4 cases in the control group, mainly gastric pain, flatulence, diarrhea, and nausea. Gao et al. [54] observed mild abdominal pain and discomfort in 1 patient, and increased stool frequency in 1 patient.

### Gut microbiota-based therapies for JIA

Two RCTs reported the treatment of JIA with probiotics. Malin et al. [64] found that probiotics increased the number of immune cells secreting IgA and IgM, and decreased fecal urease activity associated with mucosal tissue damage (*P*<0.05). Shukla et al. [63] found that probiotics may reduce IL-10 levels (*P* < 0.01) with a safety comparable to placebo. The most common adverse events were diarrhea (36% in experiment group v.s. 45% in control group), abdominal pain (9% in experiment group v.s. 20% in control group), mild infection (4.5% in experiment group v.s. 20% in control group), and flatulence (23% in experiment group v.s. 15% in control group).

### Gut microbiota-based therapies for spondyloarthritis

Two RCTs reported the results of gut microbiota-based therapies in the treatment of spondyloarthritis. The study by Jenks et al. [65] showed that compared with placebo, there was no significant difference in BASFI and BASDAI in the probiotic group compared with placebo (*P*>0.05), and the incidence of adverse events was comparable to placebo (43.8% in experiment group v.s. 38.7% in control group). Brophy et al. [66] found no significant differences in general health, gut symptoms, or severity of arthritis in the probiotic group compared with the control group (*P*>0.05). There were also no significant differences in the incidence of adverse events between the two groups (54.5% in experiment group v.s. 45.5% in control group). However, they use the Internet to recruit patients, send drugs to patients by mail, and finally obtain patient feedback through the Internet, so credibility needs to be considered.

### Gut microbiota-based therapies for psoriasis

#### PASI score

Three RCTs reported analyzable data on PASI score. Lu showed that after gut microbiota-based therapy intervention, PASI improved compared to the control group (*P*<0.05) [67]. Moludi et al. in 2021 observed that gut microbiota-based therapy significantly reduced PASI scores compared to the control group (*P*<0.05) [68]. Navarro-López et al. also reported that the improvement of PASI in the experimental group was better than that in the placebo group (*P*<0.05) [70].

#### Inflammatory factor and serum electrolytes and trace elements

Two RCTs reported CRP and IL-6 levels after probiotic treatment. Groeger et al. [69] and Moludi et al. [68] found that CRP was lower in the probiotic group compared to the control group (*P*<0.05). Moludi et al. [68] found that compared with the control group, IL-6 in the probiotic group decreased (*P*<0.05), while Groeger et al. [69] found no significant difference in IL-6 between the two groups (*P*>0.05). In addition, Groeger et al. [69] also reported TNF-α levels and showed a decrease after probiotic intervention (*P*<0.05).

Akbarzadeh et al. [71] reported serum electrolytes and trace elements. Akbarzadeh et al. [71] found that serum iron, zinc, phosphorus, magnesium, calcium, and sodium levels were significantly increased after probiotic treatment, suggesting that probiotics may alleviate mineral absorption in patients with psoriasis.

#### Adverse events of gut microbiota-based therapies for psoriasis

Two RCTs reported adverse events. Moludi et al. [68] observed gastrointestinal reactions in 12% and 8% of patients in the placebo and experimental groups, respectively. These all imply that patients tolerated probiotics well. Navarro-López et al. [70] showed a low incidence of adverse events.

### Gut microbiota-based therapies for fibromyalgia syndrome

Three RCTs reported the results of probiotics in fibromyalgia syndrome. Rao et al. [75] reported BDI and BAI, and they found that patients taking probiotics also had significantly less anxiety symptoms compared to controls (*p* = 0.01), suggesting the presence of a gut-brain interface. The other two RCTs reported meta-analyzable data, so a meta-analysis was performed.

### VAS

Two RCTs reported VAS. Roman et al. [72, 73] found that gut microbiota-based therapy did not seem to improve VAS compared with the control group (*P*>0.05). Calandre et al. [74] also showed that gut microbiota-based therapy did not improve VAS compared with the control group (*P*>0.05).

#### Fibromyalgia Impact Questionnaire (FIQ)

Two RCTs reported FIQ. Roman et al. [72, 73] found that gut microbiota-based therapy did not seem to improve FIQ compared with the control group (*P*>0.05). Calandre et al. [74] also showed that gut microbiota-based therapy did not improve FIQ compared with the control group (*P*>0.05).

#### Adverse events of gut microbiota-based therapies for fibromyalgia syndrome

Only Calandre et al. [74] reported adverse events. They reported that seven patients in the experimental group and 6 patients in the placebo group discontinued treatment due to adverse events. The vast majority of adverse events were related to the gastrointestinal tract, but there was no significant difference in the incidence of adverse events between the two groups. More RCTs are needed in the future to determine the occurrence of adverse events.

### Gut microbiota-based therapies for PSS

Only one RCT reported gut microbiota-based therapies for PSS. Kamal et al. [76] treat patients with *Lactobacillus acidophilus*, *Lactobacillus bulgaricus*, *Streptococcus thermophilus*, and *Bifidobacterium bifidum* for 5 weeks. They found a significant reduction in candida burden from baseline to week 5 in the probiotic group, while the placebo group had no statistically significant change in concomitant candida burden. The RCT has no record of adverse events, either because adverse events were not monitored or it was possible that adverse events were monitored but there were no adverse events.

### Gut microbiota-based therapies for MS

A total of 4 RCTs reported the gut microbiota-based therapies for MS. Tamtaji et al. [80] found that probiotic supplementation downregulated IL-8 and TNF-α mRNA expression in peripheral blood mononuclear cells compared with placebo. The other two RCTs reported meta-analyzable data, so a meta-analysis was performed.

#### EDSS

**Fig. 6:**
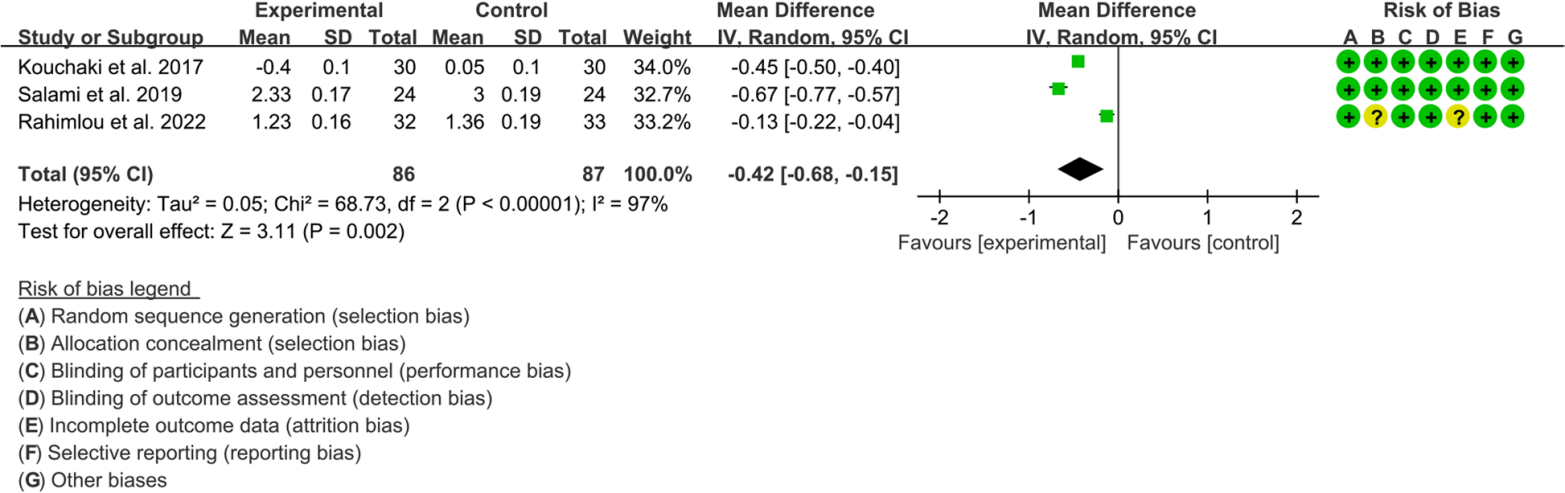
Outcomes of MS: EDSS

Three RCTs reported analyzable data on EDSS. The heterogeneity test indicated that the heterogeneity among the included RCTs was high (*I*^2^=97%, *P*<0.00001). The results of meta-analysis showed that compared with control group, the EDSS in experimental group was lower {WMD=−0.42, 95%CI [−0.68, −0.15], *P*=0.002} (Fig. 6).

#### CRP

Two RCTs reported CRP. Kouchaki et al. [77] found that CRP decreased after gut microbiota-based therapy compared with the control group (*P* = 0.01). Salami et al. [78] also showed that CRP decreased after gut microbiota-based therapy (*P* = 0.03)

#### Adverse events of gut microbiota-based therapies for MS

Only 1 RCT reported adverse events. Rahimlou et al. [79] showed that only 1 patient in the placebo group was excluded due to complaints of skin sensitivity, and none of the remaining patients experienced any serious adverse events.

### Gut microbiota-based therapies for systemic sclerosis

A total of 3 RCTs reported the gut microbiota-based therapies for systemic sclerosis. García-Collinot et al. [83] found that probiotics improved patients with gastrointestinal symptoms such as diarrhea, abdominal pain, and gas/bloating/bloating. The other two RCTs reported meta-analyzable data, so a meta-analysis was performed.

#### Total GIT

Two RCTs reported GIT. Low et al. [81] showed that although the difference of total GIT score between gut microbiota-based therapy treatment and control group was of no statistical significance (*P*= 0.85), GIT reflux was significantly improved in the gut microbiota-based therapy group (*P* = 0.004). Marighela et al. [82] also showed that compared with the control group, there was no significant difference in GIT scores in the gut microbiota-based therapy group (*P*>0.05).

#### HAQ-DI

Two RCTs reported HAQ-DI. Low et al. [81] showed that the difference of HAQ-DI between gut microbiota-based therapy treatment and control group was of no statistical significance (*P* = 0.66). Marighela et al. [82] also showed that compared with the control group, there was no significant difference in HAQ-DI in the gut microbiota-based therapy group (*P*>0.05).

#### Adverse events of gut microbiota-based therapies for systemic sclerosis

Two RCTs reported adverse events. Marighela et al. [82] did not monitor associated adverse events. García-Collinot et al. [83] showed no serious adverse events, the main adverse event being gastrointestinal symptoms; adverse symptoms occurred more frequently in the metronidazole group than in the probiotic group (22% in *S. boulardii* group v.s. in 53% Metronidazole group v.s. in 36% *S. boulardii* + Metronidazole group).

### Gut microbiota-based therapies for T1DM

A total of 4 RCTs reported the gut microbiota-based therapies for T1DM. The other RCTs reported meta-analyzable data; hence, the meta-analysis was performed.

#### HbA1c

**Fig. 7:**
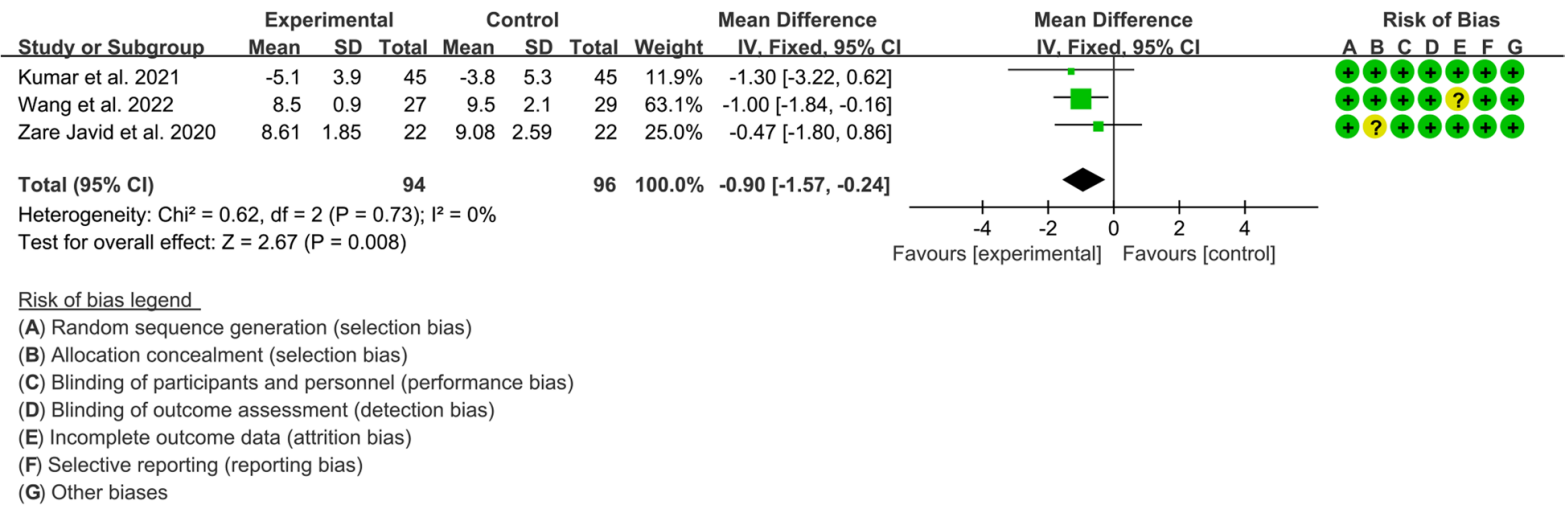
Outcomes of T1DM: HbA1c

Three RCTs reported analyzable data on HbA1c. The heterogeneity test indicated that the heterogeneity among the included RCTs was low (*I*^2^=0%, *P*=0.73). The results of meta-analysis showed that compared with control group, the HbA1c in experimental group was lower {WMD=−0.90, 95%CI [−1.57, −0.24], *P*=0.008} (Fig. 7).

#### Total insulin requirement

Two RCTs reported total insulin requirement. Kumar et al. [84] showed that compared with placebo group, the total insulin requirement decreased after gut microbiota-based therapy treatment (*P*= 0.037). However, Groele et al. [86] showed that the difference of the total insulin requirement between gut microbiota-based therapy treatment and control group was of no statistical significance (*P*= 0.619).

#### Adverse events of gut microbiota-based therapies for T1DM

A total of 2 RCTs reported the adverse events. Kumar et al. [84] suggested that this drug was well tolerated. Two patients in the experimental group reported minor adverse events such as bloating and flatulence (2 cases). Patients on the placebo had no complaints throughout the study. Groele et al. [86] did not report any adverse events.

### Gut microbiota-based therapies for OLP

#### OLP severity score

Two RCTs reported OLP severity score. Keller and Kragelund [88, 89] showed that the difference of OLP severity score between gut microbiota-based therapy treatment and control group was of no statistical significance (*P*>0.05). Li et al. [90] also showed that the difference of OLP severity score between gut microbiota-based therapy treatment and control group was of no statistical significance (*P*>0.05).

#### Adverse events of gut microbiota-based therapies for OLP

Only Li et al. [90] reported adverse events. They did not observe any adverse events in their research.

### Gut microbiota-based therapies for Crohn’s disease

A total of 3 RCTs reported probiotic treatment of Crohn’s disease. Since the indicators of RCTs are not uniform, only a systematic review was conducted. Yılmaz et al. [91] found that ESR and CRP were significantly decreased after probiotic intervention, while hemoglobin was increased, and within the past 2 weeks, abdominal distension scores were significantly decreased and feeling good scores increased (*P*<0.05). Schultz et al. [92] showed that 5 patients completed the study, and 2 patients in both the probiotic and control groups had sustained remission. The median time to relapse was 16 ± 4 weeks in the probiotic group and 12 ± 4.3 weeks in the placebo group. Steed et al. [93] found that the Crohn’s disease activity index and histological score were decreased in patients after synbiotic intervention (*P* < 0.05), but synbiotics had little effect on mucosal IL-18, INF-γ, and IL-1β. However, TNF-α expression was significantly decreased in the synbiotic group at 3 months (*P*<0.05), but not at 6 months. Those RCT has no record of adverse events, either because adverse events were not monitored or it was possible that adverse events were monitored but there were no adverse events.

### Gut microbiota-based therapies for ulcerative colitis

#### Endoscopy score

**Fig. 8:**
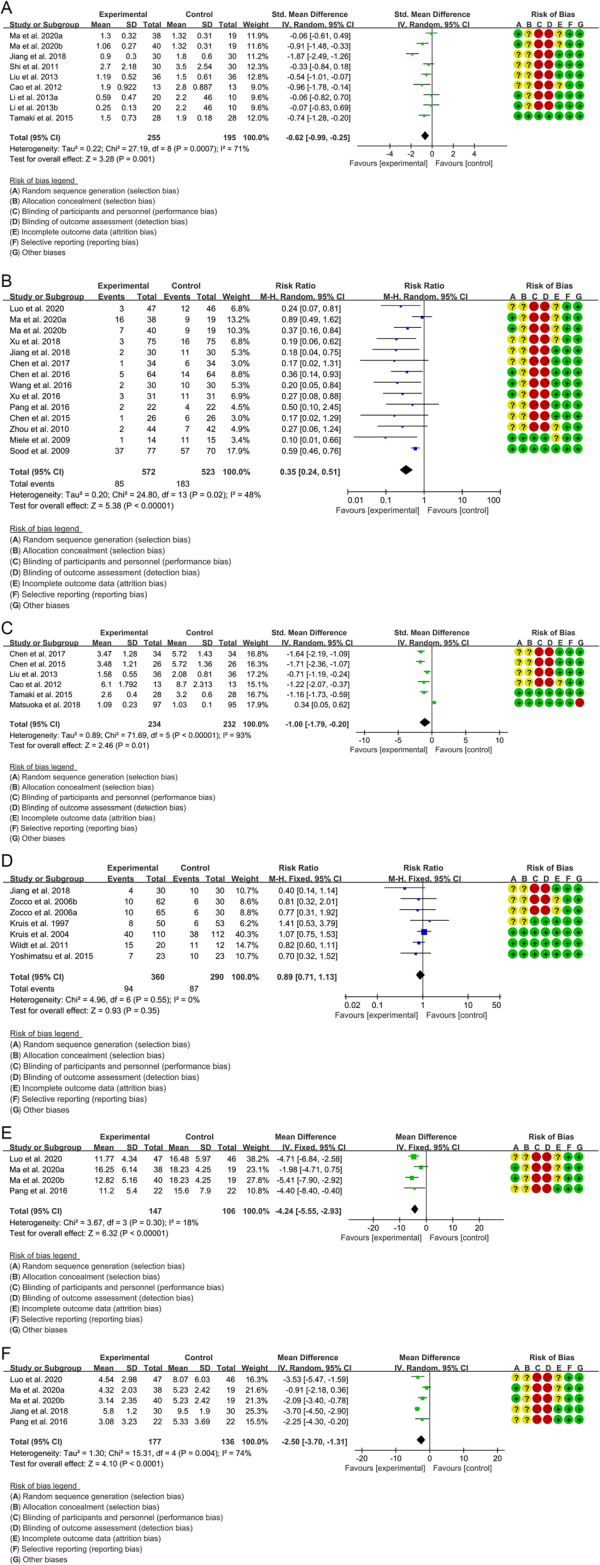
Outcomes of ulcerative colitis (**A** Endoscopy Score; **B** Ineffective rate; **C** Disease activity; **D** Relapse rate; **E** ESR; F: CRP)

Seven RCTs reported analyzable data on endoscopic scores. The heterogeneity test indicated that the heterogeneity among the included RCTs was high (*I*^2^=71%, *P*=0.0007). The results of meta-analysis showed that compared with control group, the endoscopy score in experimental group was lower {SMD=−0.62, 95%CI [−0.99, −0.25], *P*=0.001} (Fig. 8A). The publication bias test result showed *P*=0.94, suggesting that there may be no publication bias (see supplementary materials figure: Additional file 4).

#### Ineffective rate

Thirteen RCTs reported analyzable data on ineffective rate. The heterogeneity test indicated that the heterogeneity among the included RCTs was high (*I*^2^=48%, *P*=0.02). The results of meta-analysis showed that compared with control group, the endoscopy score in experimental group was lower {RR=0.35, 95%CI [0.24, 0.51], *P*<0.00001} (Fig. 8B). The publication bias test result showed *P*=0.012, suggesting the possibility of publication bias (see supplementary materials figure: Additional file 5).

#### Disease activity

Six RCTs reported available disease activity, and the SMD was used to express the effect size because different criteria were used. The heterogeneity test indicated that the heterogeneity among the included RCTs was high (*I*^2^=93%, *P*<0.00001). The results of meta-analysis showed that compared with control group, the disease activity in experimental group was lower {SMD=−1.00, 95%CI [−1.79, −0.20], *P*=0.01} (Fig. 8C). The publication bias test result showed *P*=0.013, suggesting the possibility of publication bias (see supplementary materials figure: Additional file 6).

#### Relapse rate

Six RCTs reported analyzable data on relapse rate. The heterogeneity test indicated that the heterogeneity among the included RCTs was low (*I*^2^=0%, *P*=0.55). The results of meta-analysis showed that the relapse rate between experimental group and control group was of no statistical significance {RR=0.89, 95%CI [0.71, 1.13], *P*=0.35} (Fig. 8D). The publication bias test result showed *P*=0.198, suggesting that there may be no publication bias (see supplementary materials figure: Additional file 7).

#### ESR

Three RCTs reported analyzable data on ESR. The heterogeneity test indicated that the heterogeneity among the included RCTs was low (*I*^2^=18%, *P*=0.30). The results of meta-analysis showed that compared with control group, the ESR in experimental group was lower {WMD=−4.24, 95%CI [−5.55, −2.93], *P*<0.00001} (Figure 8E).

#### CRP

Four RCTs reported analyzable data on CRP. The heterogeneity test indicated that the heterogeneity among the included RCTs was high (*I*^2^=74%, *P*=0.004). The results of meta-analysis showed that compared with control group, the CRP in experimental group was lower {WMD=−2.50, 95%CI [−3.70, −1.31], *P*<0.0001} (Fig. 8F).

#### Adverse events of gut microbiota-based therapies for ulcerative colitis

**Fig. 9:**
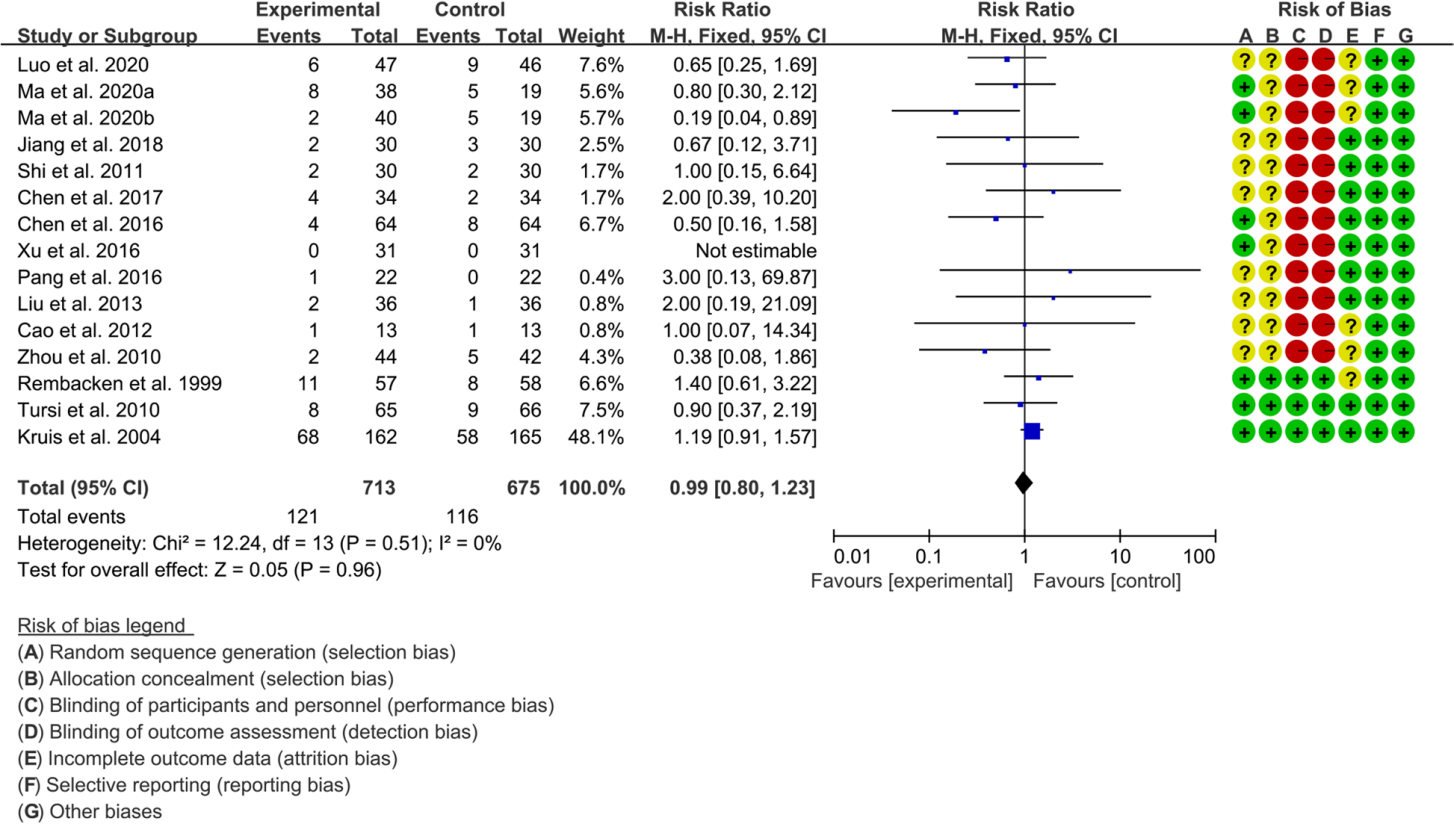
Adverse events of gut microbiota-based therapies for ulcerative colitis

A total of 24 RCTs reported the adverse events. Fourteen (14) RCTs reported the number of adverse events and were therefore pooled for meta-analysis. The results of heterogeneity analysis showed that the heterogeneity between groups was low, and a fixed effect model was used (*I*^2^=0%, *P*=0.51). The results showed that the adverse events between experimental group and control group was of no statistical significance {RR=0.99, 95%CI [0.80, 1.23], *P*=0.96} (Fig. 9). Kato et al., Matthes et al., Sánchez-Morales et al., Sood et al., Tamaki et al., Kruis et al., Matsuoka et al., Wildt et al., and Yoshimatsu et al. reported that no significant adverse events were observed. The publication bias test result showed *P*=0.404, suggesting that there may be no publication bias (see supplementary materials figure: Additional file 8).

#### Sensitivity analysis of gut microbiota-based therapies for ulcerative colitis

The number of RCTs in 3 outcomes was >5 and their heterogeneity was high, so sensitivity analysis was performed. For endoscopy score and ineffective rate, no matter which RCTs are eliminated, it has little impact on the overall results, indicating that the results are stable (see supplementary materials figure: Additional file 9). For disease activity, the results changed significantly after removing Matsuoka et al. [120] [64], suggesting that Matsuoka et al. [120] may be the source of heterogeneity (see supplementary materials figure: Additional file 9).

## Discussion

Overall, this systematic review and meta-analysis found that gut microbiota-based therapies may improve the symptoms and inflammatory factor of celiac sprue, SLE and LN, JIA, psoriasis, fibromyalgia syndrome, PSS, MS, T1DM, Crohn’s disease, and ulcerative colitis, but may not improve the symptoms and/or inflammatory factor of spondyloarthritis and RA. From Table S2, it can be found that almost all treatments in RCTs are based on *Bifidobacteria* and *Lactobacilli*; hence, gut microbiota-based therapy based on *Bifidobacteria* and *Lactobacilli* may be an effective and safe therapy for these autoimmune diseases. The mechanism is discussed as follows:

### Pathological mechanisms of gut microbiota in autoimmune diseases

Autoimmune diseases refer to the process in which the body’s immune dysfunction reacts to autoantigens. In the case of immune disorders, the body will attack autoantigens and cause a series of immune responses. In the immune process, it will cause organ damage and a series of clinical symptoms, causing organ damage and leading to clinical diseases [123–125]. At present, nearly 100 kinds of autoimmune diseases have been found in the world. Common autoimmune diseases include RA, SLE, ulcerative colitis, MS, and so on. Such diseases are more common in women, the global incidence rate of about 0.09%, an upward trend year by year [126, 127]. The manifestations of autoimmune diseases are clinically heterogeneous and the pathogenesis is complex [128]. Recent studies have shown that in addition to abnormal immune tolerance, the pathogenesis of autoimmune diseases may also be related to genetic susceptibility, environmental incentives, and intestinal flora imbalance. In particular, intestinal flora and increased intestinal permeability are involved in the imbalance of innate immunity and adaptive immunity in autoimmune diseases [129, 130].

**Fig. 10:**
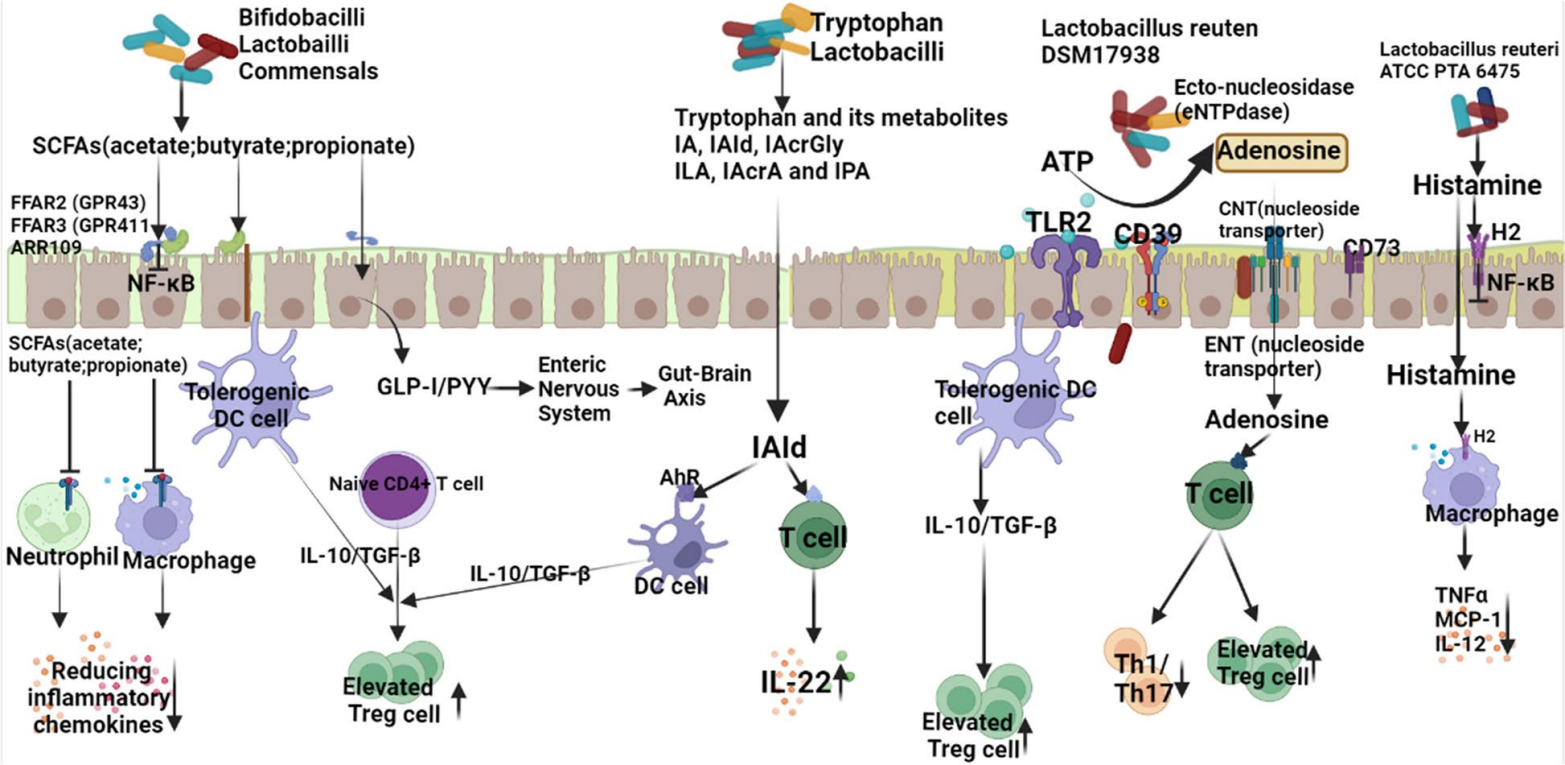
Potential molecular mechanism of probiotics in the treatment of autoimmune diseases (The mechanism is summarized from [11, 26, 27, 129, 136–139, 151–158]. AhR: aryl hydrocarbon receptor; CNS: central nervous system; FFARs: free fatty acid receptors; GLP1: glucagon-like protein-1; GPRs: G-binding protein receptors; H2: histamine receptor 2; PYY: peptide tyrosine tyrosine; SCFAs: short-chain fatty acids)

Current studies have shown that to a certain extent, the intestinal barrier and the human immune system have a complex two-way effect [131, 132]. The intestinal barrier is mainly composed of intestinal commensal bacteria, intestinal mucus layer, intestinal epithelial cells, and various immune cells in the lamina propria, such as dendritic cells (DC), T cells, and B cells [133]. When the body is in a steady state, the gut microbiota and the host maintain a dynamic equilibrium relationship. When the pathogen invades, this balance will be broken, and it will mistakenly identify and attack its own tissues, triggering the body’s autoimmune disease. Therefore, autoimmune diseases often appear immune disorders of innate immunity and adaptive immunity [134, 135]. Innate immunity is a defense system against pathogens at the genetic level, and the flora can promote the production of related cytokines by activating innate immune cells such as macrophages or DCs [136]. Some studies suggest that *B. fragilis* has the ability to induce the phagocytes of the lamina propria to produce the anti-inflammatory cytokine IL-10, thereby activating Treg and increasing immune regulation [137]. Another study found that the adhesion of segmented filamentous bacteria (SFB) to the host can upregulate the level of serum amyloid A (SAA). It promotes the production of IL-6 and IL-23 through CD11c* lamina propria DCs and can induce the proliferation and differentiation of Th-17 in the small intestine to play an anti-infective role, which may be related to the occurrence of autoimmune diseases [138]. In addition, SFB and intestinal epithelial cells may stimulate the production of reactive oxygen species, increase the secretion of IL-Iβ, and promote the differentiation of Th17 cells [139]. Studies have shown that natural killer (NK) cells detect and clear transformed and infected target cells by producing IFN-c or perforin, and gut microbiota may play a key role in promoting IL-22+NKp46+ cell differentiation [140]. Among them, neutrophils in GF were significantly reduced, and NKp46+ cells that produced IL-22 were also lacking [141]. Adaptive immunity, also known as acquired immunity, is formed after the stimulation of antigenic substances such as microorganisms and can react specifically with the antigen [142]. Intestinal flora is involved in adaptive immunity, which can promote the production of IgA in the gut by stimulating B cell responses, and can also accelerate inflammatory responses or affect immune tolerance by regulating T cell differentiation [143]. CD4+ T cells are an important component of the adaptive immune response, including four subtypes of Th1, Th2, Th17, and Treg. Among them, Th1 and Th17 play an important role in the process of autoimmunity, and Treg is a key mediator of immune tolerance [144]. Toll-like receptors (TLRs) act as pattern recognition receptors to eliminate pathogens by recognizing distinct but overlapping microbial components [145]. Removal of TLR2 from the surface of CD4+ T cells leads to an antimicrobial immune response, which reduces the number of *B. fragilis* [146]. SCFAs can directly promote the differentiation of naive T cells to Th1 and Th17 [147] and may also increase the expression of forkhead-like transcription factor 3 in colonic T cells by activating G protein-coupled receptor 43 antibodies on T cells [148], which in turn triggers inflammation. Butyrate (one of SCFAs) can regulate the differentiation of T lymphocytes in the intestinal tract and then play an anti-inflammatory effect [149]. In summary, the gut microbiota ecology of patients with autoimmune diseases is out of balance, and some types of microorganisms are associated with key clinical indicators or disease subtypes of specific autoimmune diseases. Their increase or decrease indicates their potential pro-inflammatory or anti-inflammatory effects, and more importantly, changes in metabolic function mediated by gut microbiota. Abnormal synthesis pathway or degradation pathway caused by intestinal flora imbalance can lead to intestinal ecological destruction and pathological damage [150]. For example, recent studies have shown that dysbiosis of the gut microbiota, characterized by a reduction in *Bifidobacterium*, is associated with increased disease activity in patients with autoimmune hepatitis [151]. By evaluating the disease stages of different autoimmune hepatitis patients, probiotics may be considered as an adjuvant therapy for non-responsive autoimmune hepatitis in the future, aiming to prevent recurrent deterioration and disease progression in these patients [152]. The specific mechanism of intestinal flora can be seen in Fig. 10.

In 1965, probiotics were proposed, whose role is to promote the reproduction of beneficial bacteria, inhibit the growth of pathogenic bacteria, maintain the balance of intestinal flora, and benefit human health [153]. At present, probiotics have been widely used in the treatment of gastrointestinal diseases. Commonly used probiotics are *Lactobacillus*, *Bifidobacterium*, *Acidophilus*, etc. [154]. In addition, probiotics can also play a role in immune regulation [155]. *Lactobacillus* and *Bifidobacterium* are important anti-inflammatory bacteria in probiotics. *Lactobacillus casei* can increase anti-inflammatory cytokines (such as IL-10, TGF-β) and inhibit pro-inflammatory cytokines (such as IL-1β, IL-2) [154], while *Lactobacillus plantarum* LC27 and *Bifidobacterium longum* LC67 can inhibit NF-κB pathway to inhibit inflammatory response [155]. In addition, *Lactobacillus casei* can promote the differentiation of CD4+ T cells into Treg and inhibit their differentiation into Th17 cells, thereby regulating immune function [156, 157]. In summary, the pathogenesis of autoimmune diseases is closely related to intestinal flora, probiotics may alleviate autoimmune diseases by correcting intestinal flora imbalance, improving intestinal microecology, increasing intestinal wall compactness, inhibiting the translocation of bacteria and their metabolites, thereby inhibiting pro-inflammatory signaling pathway, regulating CD4+ T cell differentiation, and inhibiting the production of pro-inflammatory factors [158]. Probiotics play an important role in regulating intestinal microecological balance and regulating the immune function of the body, which makes them have broad application prospects in the field of autoimmune disease treatment [27]. The potential molecular mechanism of probiotics in the treatment of autoimmune diseases is shown in Fig. 10. Probiotics are undoubtedly the hope of autoimmune patients with poor response to conventional treatment or with adverse reactions. Due to the variety and complex characteristics of probiotics, their mechanism of action is difficult to define and needs to be further explored. This systematic review and meta-analysis comprehensively summarized the clinical research of probiotics on autoimmune diseases, in order to provide a firm basis for clinical treatment of various autoimmune diseases.

### Gut microbiota-based therapies for celiac sprue

Celiac disease is an autoimmune intestinal disease induced by gluten intake in genetically susceptible individuals, which can cause pathological changes such as infiltration of small intestinal mucosal intraepithelial cells, crypt hyperplasia, and villous atrophy [159]. However, there are other factors that influence the development of celiac disease [160]. Studies have found that the duodenum of patients with celiac disease is dominated by Gram-negative bacteria and contains more pro-inflammatory bacteria [161], and the abundance of *Proteobacteria* and their genera increased, and the ratio of *Bifidobacterium*/*Neisseria* decreased [162]. Further research found that a variety of commensal bacteria in the intestinal tract of patients with celiac disease carry a large number of virulence genes, suggesting that their symbiotic relationship with the host may be altered. A study using comparative genomics analysis method found for the first time that *Nesterenkonia jeotgali*, which is enriched in the gut of celiac disease patients, contains more genes related to iron uptake, antibiotic resistance, and oxidative stress [163]. There are also significant differences in the metabolic characteristics of the gut microbiota between celiac disease patients and healthy controls [164]. Recent research indicates that increased intestinal permeability exacerbates the dysregulation and imbalance of the immune system in response to the heightened interaction between immune cells and the gut microbiota. Evidence suggests that over half of untreated celiac disease patients exhibit antibodies against S protein, and irrespective of the severity of mucosal damage, there is a positive presence of cerevisiae. The presence of cerevisiae in celiac disease suggests its potential impact on nonspecific immune responses during the course of chronic small intestinal diseases [165]. Regardless of whether a gluten-free diet is taken, the propionic acid content in the feces of celiac disease patients is always higher than that of healthy controls, which may be due to the increased abundance of propionic acid-producing bacteria in the gut [166]. When the intestinal permeability or the metabolic state of the flora changes, a large amount of volatile organic compounds (VOCs) are produced. VOCs can enter body fluids and can be detected in blood, urine, or sweat. The researchers identified 15 biomarkers by comparing the characteristics of urinary VOCs in patients with celiac disease and healthy people [167]. In terms of altering the structure and function of the intestinal barrier, studies have shown that *Shigella* and *Escherichia coli* isolated from the intestinal tract of patients with celiac disease can induce intestinal tight junction damage by adhering to intestinal mucosal epithelial cells, and this damage may be related to metalloproteinases [168]. In addition, due to the imbalance of gut microbiota and the disruption of gut barrier function, a variety of opportunistic pathogens may directly contact host cells to regulate gut immune responses to gluten. For example, *Neisseria flavescens* isolated from the duodenum of patients with celiac disease can escape the degradation of lysosomes in Caco-2 cells and directly induce dendritic cells to release inflammatory factors such as INF-γ and TNF-α [169]. The direct interaction between the flora and the host is mostly mediated by the Toll-like receptor (TLR) family. Compared with healthy controls, celiac disease patients had increased TLR4 mRNA expression in peripheral blood, while TLR2 and TLR4 mRNA expression was decreased in duodenal biopsy specimens. TLR4 can recognize lipopolysaccharide, the main component of the cell wall of Gram-negative bacilli, activate the TLR4/MyD88/NF-κB signaling pathway, and promote the production of inflammatory factors [170]. This systematic review also suggested that probiotic preparations may improve the intestinal flora and reduce the level of TNF-α in patients with celiac disease. The included RCTs also showed that probiotics can reduce tTGA levels, modulate peripheral immune responses, etc.

### Gut microbiota-based therapies for SLE and LN

SLE is an autoimmune disease involving multiple systems, multiple organs, and the appearance of multiple autoantibodies. The pathogenesis of SLE is very complex and unclear, but a large number of studies have confirmed that the dysregulation of T lymphocytes in the circulation of SLE patients is one of the characteristics of SLE, and its severity is related to disease activity [171]. Further research found that T cell dysregulation is caused by APC function defect, and DC is the most powerful APC in the body, so DC function defect is the main cause of T cell function defect [172]. In addition, the abnormal expression of cytokines IL-1, IL-6, TNF-α, IFN-γ, IL-4, and IL-10 in SLE patients further revealed that immune cells and cytokines mediate the occurrence of SLE [173].

Studies have found that SLE patients have intestinal flora imbalance. It is characterized by a significant decrease in *Firmicutes/Bacteroidetes* ratio, a decrease in intestinal flora diversity, an increase in the number of Gram-negative bacteria, and an increase in serum lipopolysaccharide (LPS) [174]. Patricia et al. incubated naive T cells with inactivated fecal flora from SLE patients and healthy people, respectively, and found that the former can more promote the differentiation of Th17 cells. Appropriate supplementation of *Bifidobacterium bifidum* LMG13195 promotes Foxp3 expression and enables naive CD4+ T cells to develop into Treg rather than Th17 cells [175]. Compound probiotics (*Lactobacillus rhamnosus* and *Lactobacillus del brueckii*) prophylactically fed SLE model mice for 2 months, the levels of related autoantibodies and the frequency of Th1 and Th17 cells in the spleen were decreased; meanwhile, the serum pro-inflammatory factors IL-17 and IFN-γ levels decreased [176]. Meanwhile, probiotic *Lactobacillus fermentum* CECT5716 can regulate intestinal microecology, increase intestinal density, reduce LPS in serum, restore Th17/Treg balance, and inhibit vascular endothelial oxidative stress [177]. Therefore, probiotics can be considered as adjunctive therapy to prevent vascular complications of SLE.

In terms of regulating DC cells and Treg cells, Hsu et al. evaluated the intervention effect of *Lactobacillus paracasei* GMNL-32, *Lactobacillus reuteri* GMNL-89, and *Lactobacillus reuteri* GMNL-263 on animal models of systemic lupus erythematosus [178]. They found that *Lactobacillus* can alleviate SLE-related symptoms, the possible mechanism is by inhibiting NF-κB pathway and extracellular signal-regulated kinase inflammatory pathway, thereby reducing the expression of TNF-a, IL-1β, increasing the expression of anti-inflammatory cytokines IL-10, so as to reduce inflammation. This meta-analysis also showed that probiotics may reduce SLEDAI scores, and reduce Complement C3 and IgG levels, and are relatively safe.

### Gut microbiota-based therapies for RA

RA is a chronic disease accompanied by symptoms such as joint pain, hyperalgesia, edema, and irreversible destruction of bone and cartilage, resulting in joint deformity, which may lead to disability if not treated in time [179]. The pathogenesis of RA is still unclear, but intestinal flora disturbance is considered to be the trigger for the occurrence of RA [11]. Studies have found that the fecal flora of RA patients is significantly different from that of healthy subjects. Compared with healthy people, the content of probiotics such as *Bifidobacterium*, *Bacteroides*, and *Lactobacillus* in the intestinal flora of RA patients is significantly lower, while the content of *Escherichia coli* and *Enterococcus* is significantly higher [180, 181]. In RA, adaptive immunity, dominated by CD4+ T cells, plays an important role in initiating and maintaining the autoimmune response characteristic of rheumatoid arthritis [182]. Fan et al. found that *Lactobacillus* can reduce the expression of cytokines IL-12, IFN-γ, TGF-β, and IL-6 in collagen-induced arthritis (CIA) mice, induce Th1 and Th17 cell differentiation, and improve intestinal microbiota imbalance [183]. Based on the above results, it is speculated that early intervention of probiotics is more conducive to clinical relief of RA symptoms. RA symptoms are closely related to the excessive production of pro-inflammatory factors and the activation of intracellular pro-inflammatory signals. Shadnoush et al. found that the joint swelling and pain sensitivity of CIA mice were weakened, and the infiltration of inflammatory cells was reduced after the intervention of different doses of probiotics (*Bifidobacterium breve*, *Lactobacilluscasei*, *Lactobacillus bulgaricus*, *Lactobacillus rhamnosus*, and *Lactobacillus acidophilus*). And serum IL-1β levels decreased, spinal cord activation of p38 mitogen-activated protein kinase (MAPK) inflammatory pathway was inhibited. p38MAPK is an important inflammatory signaling pathway in cells [184]. Intestinal microbes reduce inflammatory factors by regulating redox balance may be one of the mechanisms of probiotics to alleviate RA [185]. This meta-analysis did not show an improvement in DAS28 and joint symptoms with probiotics; however, some RCTs showed good efficacy. For example, Zamani et al. found that after taking probiotic capsules for 8 weeks in RA patients, compared with the placebo group, serum C-reactive protein and insulin levels decreased; DAS28 scores decreased, indicating that the disease was significantly improved [57]. More RCTs are needed in the future to revise the results.

### Gut microbiota-based therapies for JIA

JIA is a common connective tissue disease in children, characterized by chronic joint synovitis, and is one of the main diseases that lead to disability and blindness in children [186, 187]. The treatment goal of JIA is to relieve the clinical symptoms of children to the greatest extent, prevent and reduce the adverse reactions of organ damage and treatment, so as to improve the quality of life of children [188, 189]. It is considered to be multifactorial, with a strong interaction between genetic susceptibility and environmental triggers [190]. The innate immune system appears to play a central role in the pathogenesis of systemic JIA (SoJIA) [191]. In contrast, other forms of JIA are generally thought to be driven by T cells and are generally associated with increases in pro-inflammatory cytokines such as tumor necrosis factor, IL-1, and IL-6. However, T helper 17 (Th17) cells secreting the pro-inflammatory cytokines IL-17 and IL-22 have recently been implicated in the pathogenesis of JIA [188, 192]. In JIA patients, these pro-inflammatory responses can be counteracted by specialized T cells called IL-10-producing regulatory T cells (Tregs) [193]. In recent years, the gut microbiota has gradually become an important factor in the pathogenesis of JIA, and several comparative studies have shown that changes in the gut microbiota may be the cause of the disease pathogenesis [194–196]. At the phylum level, *Bacteroidetes*/*Bacteroidetes* are reported to have increased abundance in JIA patients [197–200]. At the genus level, *Bacteroidetes* increased in JIA patients and revealed a significant decrease in *Firmicutes* [201]. In summary, abnormal gut microbiota may influence the development of JIA by mediating host immune programs and altering mucosal permeability. Gut microbiota dysbiosis may contribute to the dysregulation of the immune system by regulating the development of T cell subsets, especially Th17 cells and Treg, and by increasing mucosal permeability. Combined with host genetic susceptibility and environmental triggers, gut microbiota dysbiosis may lead to autoimmunity and local inflammation in extraintestinal sites such as joints [202, 203].

In this systematic review, two RCTs reported the treatment of JIA with probiotics. Malin et al. found that probiotics increased the number of immune cells secreting IgA and IgM, and decreased fecal urease activity associated with mucosal tissue damage (*P*<0.05) [64]. Shukla et al. found that probiotics may reduce IL-10 levels (*P* < 0.01) with a safety comparable to placebo. The most common adverse events were diarrhea (36% in experiment group v.s. 45% in control group), abdominal pain (9% in experiment group v.s. 20% in control group), mild infection (4.5% in experiment group v.s. 20% in control group), and flatulence (23% in experiment group v.s. 15% in control group) [63].

### Gut microbiota-based therapies for spondyloarthritis

Ankylosing spondylitis is a disease characterized by inflammation of the sacroiliac joints and spinal attachment points as the main symptom, which is strongly associated with HLA-B27. Certain microorganisms (such as *Klebsiella*) share antigens with susceptible individuals’ own tissues, which can trigger abnormal immune responses. It is a chronic inflammatory disease characterized by fibrosis and ossification of the large joints of the limbs, as well as the intervertebral annulus fibrosus and its adjacent connective tissue, as well as ankylosis. It can also involve the internal organs and other tissues. Chronic progressive rheumatic disease [204–208]. Its pathogenesis is very complex and still not fully understood. In recent years, studies have found that the imbalance of intestinal flora homeostasis can trigger the body’s inflammatory response, which is closely related to the occurrence of AS [209]. The structure of the gut microbiota in AS patients is significantly altered compared with the normal population [210, 211]. *Bifidobacterium bifidum*, *Bifidobacterium longum*, and *Bifidobacterium pseudochain* are reported to induce Th2-driven immune responses [212]. The above studies have shown that the gut microbiota is altered in AS patients, and this change may play a role by regulating the innate and adaptive immune systems. The microbiota modulates the gut immune response mainly through microbe-associated molecular patterns (MAMPs) such as LPS and flagellin. In the innate immune response, bacterial chemotaxis is attenuated due to decreased levels of LPS and flagellin, regulation of the actin cytoskeleton. FcγR-mediated phagocytosis and nodular receptor signaling-induced secretion of the antimicrobial peptide RegIIIγ can lead to dysbiosis of gut microbiota and the occurrence of AS.

In this systematic review, two RCTs reported the results of probiotics in the treatment of spondyloarthritis. The study by Jenks et al. showed that compared with placebo, there was no significant difference in BASFI and BASDAI in the probiotic group compared with placebo (*P*>0.05), and the incidence of adverse events was comparable to placebo (43.8% in experiment group v.s. 38.7% in control group) [65]. Brophy et al. found no significant differences in general health, gut symptoms, or severity of arthritis in the probiotic group compared with the control group (*P*>0.05). There were also no significant differences in the incidence of adverse events between the two groups (54.5% in experiment group v.s. 45.5% in control group) [66]. More RCTs are needed in the future to revise the results.

### Gut microbiota-based therapies for psoriasis

Psoriasis is a chronic inflammatory skin disease with a long course of disease and a tendency to recur easily, and in some cases it is almost lifelong. The clinical manifestations of the disease are mainly erythema and scales, which can occur all over the body, and are more common on the scalp and extensor limbs [213]. Psoriasis may be related to genetic factors, immune dysfunction, and environmental factors. It is clinically divided into psoriasis vulgaris, interstitial psoriasis, erythrodermic psoriasis, pustular psoriasis, and joint psoriasis [214]. Studies have shown that psoriasis is mainly associated with the Th cell 17/IL-23 axis and that the gut microbiota can participate in the differentiation of T cells [199, 215, 216]. Experiments have shown that gut flora can affect the differentiation of primitive T cells, and the differentiated Treg cells can inhibit Th17 cells from attacking pathogens, which are potential pathogens and usually act as symbionts of healthy individuals [217]. In T cell-mediated inflammation, SCFA-producing microbiota and SCFAs are effective regulators of T cells [212, 218, 219]. Among them, symbiotic *Clostridium* is the main producer of SCFAs, which can induce the production of IL-10 in the colon, increase the number of Treg cells in the mucosa, and play a key role in intestinal homeostasis. [220]. As important biological macromolecules to maintain host homeostasis and control diseases, SCFAs can defend or reduce the effects of obesity, diabetes, inflammatory bowel disease, and cardiovascular disease on the body [221–223]. In summary, intestinal flora is involved in the occurrence and development of psoriasis and related comorbidities. Inflammatory cytokines can lead to changes in intestinal flora, and changes in flora also affect inflammatory cytokines, and the two interact and interact with each other.

The results of this meta-analysis show that probiotics can improve PASI scores; a systematic review shows that probiotics can improve inflammatory markers. However, due to the small number of RCTs, no firm conclusions can be drawn, and more RCTs are needed to confirm or revise the results.

### Gut microbiota-based therapies for fibromyalgia syndrome

Fibromyalgia syndrome is a chronic progressive disease characterized by extensive persistent pain in the skeletal muscles and is often accompanied by symptoms such as anxiety, depression, sleep disturbance, chronic fatigue, or gastrointestinal dysfunction in clinical practice [224, 225]. Its pathogenesis is still unclear, and there is currently no definite treatment, which seriously affects the quality of life of patients. In recent years, studies have found that fibromyalgia syndrome is associated with oxidative stress, central pain sensitization, genetic polymorphism of transporter proteins, abnormal biogenic amine content and function, excessive release of inflammatory factors, intestinal flora disturbance, or vitamin D deficiency [226–228]. Most patients with fibromyalgia syndrome have gastrointestinal disorders, of which irritable bowel syndrome (IBS) is the most common [229, 230]. The pathogenesis of fibromyalgia syndrome is mostly related to mental stress and trauma, and multi-level treatment is adopted in the treatment, including exercise, patient education and cognitive behavior, antidepressants, analgesics, and other drug treatments [231]. Fibromyalgia syndrome and IBS provide an interesting model for the relationship between gut bacteria and somatic hypersensitivity, and the ability of the gut microbiota to regulate the immune system is thought to be an important factor in the pathogenesis [232]. Changes in the number and distribution of intestinal flora in patients with fibromyalgia syndrome lead to an increase in the permeability of the intestinal barrier, which may be one of its pathogenic mechanisms [233]. Meanwhile, some scholars have studied the relationship between hyperalgesia and toxins produced by intestinal flora [234]. Whether the hyperalgesia symptoms of fibromyalgia syndrome are related to the effect of endotoxin caused by intestinal flora disturbance will require further research. Roman et al. found that the symptoms of patients with fibromyalgia syndrome improved significantly after taking gut microbiota-based therapies for 8 weeks [72, 73]. It is speculated that by affecting the central nervous system through the brain-gut axis, probiotics can promote the production and transmission of neuroactive substances, improve the intestinal epithelial barrier function, correct intestinal immune abnormalities, and reduce the production and release of pro-inflammatory cytokines.

This meta-analysis shows that probiotics can improve pain (reduce VAS) in patients with fibromyalgia syndrome and are relatively safe, but have no significant improvement in FIQ. However, due to the small number of RCTs, no firm conclusions can be drawn, and more RCTs are needed to confirm or revise the results.

### Gut microbiota-based therapies for PSS

PSS is a common autoimmune disease in which inflammatory cells infiltrate exocrine glands and extraglandular epithelium [235, 236]. It is a benign disease involving multiple factors such as genetics, environment, and hormones. It has a good prognosis, and most of them can be controlled and relieved after treatment [237]. The pathogenesis of PSS is mainly related to inflammatory cells such as plasmacytoid dendritic cells, T lymphocytes, and B lymphocytes [238]. Microbial infection of the exocrine glands results in the elevation of type 1 interferon (IFN) in plasmacytoid dendritic cells and in the apoptosis of glandular epithelial cells, exposing self-antigens to autoantibodies, and subsequently triggering autoimmunity [27, 239]. The activation of T lymphocytes and B lymphocytes can activate adaptive immunity, lead to the production of related antibodies and memory lymphocytes, and promote the infiltration of inflammatory cells into the glands, and the pro-inflammatory cytokines secreted by inflammatory cells can further lead to glandular tissue damage [236]. Intestinal dysbiosis exists in PSS patients [239, 240]. Argyropoulou et al. found that PSS patients had an increase in intestinal pathogenic bacteria and a decrease in the number of commensal bacteria [241]. De Paiva et al. found that the number of *Bacteroides*, *Parabacteria*, *Faecalibacterium*, and *Prevotella* in the intestinal flora of PSS patients decreased, while the number of *Pseudobutylicum*, *Escherichia coli*, *Shigella*, *Brucella*, and *Streptococcus* increased [242]. Van Der Meulen et al. found that the ratio of *Firmicutes* and *Bacteroidetes* in the intestinal flora of PSS patients was lower than that of the normal population, and the diversity of intestinal microbial communities in SS patients was lower than that of the normal population [243]. One study examined the function of Treg in PSS patients and found that reduced Treg inhibitory ability also played a role in the development of PSS disease [244]. The gut microbiota plays an important role in maintaining the balance of immune responses between Treg and Th17 on the mucosal surface, and acts as a trigger for autoimmune diseases such as SLE, RA, and PSS [245].

In this systematic review, only one RCT reported gut microbiota-based therapies for PSS. In Kamal et al. interventions with *Lactobacillus acidophilus*, *Lactobacillus bulgaricus*, *Streptococcus thermophilus*, and *Bifidobacterium bifidum* for 5 weeks, they found a significant reduction in candida burden from baseline to week 5 in the probiotic group, while the placebo group had no statistically significant change in concomitant candida burden. More RCTs are needed in the future to revise the results.

### Gut microbiota-based therapies for MS

MS is an autoimmune disease characterized by white matter demyelinating lesions of the central nervous system and the interaction of genetically susceptible individuals and environmental factors [246, 247]. The symptoms and histopathological features of experimental autoimmune encephalomyelitis (EAE) are highly similar to human MS, and it is the most recognized animal model of MS [248–251]. With the proposal of the brain-gut axis, the application of probiotics in neurological diseases has become more and more extensive. Gut microbiota can participate in the regulation of central nervous system function through neural, immune, and metabolic pathways [252]. After both MS patients and normal volunteers took VSL#3 (*Bifidobacterium, Lactobacillus, Streptococcus*) probiotics at the same time, the abundance of intestinal bacteria increased, while the proportion of pro-inflammatory monocytes and the expression of HLA-DR on the surface of dendritic cells (DC) decreased in MS patients [253]. Yamashita et al. found that *Lactobacillus helveticus* SBT2171 could reduce the clinical score and infiltration of spinal mononuclear cells in EAE mice and significantly inhibit Th17 cells in the inguinal lymph nodes [254]. The commercial probiotic *Lactibiane iki* reduced symptom scores in EAE mice in a dose-dependent manner and promoted the development of central nervous system myeloid dendritic cells (MDCs) towards immature immunosuppressive functions [255]. The above results show that the mechanism of probiotics preventing and alleviating the symptoms of EAE mice is mainly by inhibiting inflammatory CD4+ T lymphocytes, increasing Treg cells, and reducing the inflammatory response of the central nervous system.

This meta-analysis shows that probiotics can decrease EDSS and CRP and are relatively safe. However, due to the small number of RCTs, no firm conclusions can be drawn, and more RCTs are needed to confirm or revise the results.

### Gut microbiota-based therapies for systemic sclerosis

Systemic sclerosis is an immune-mediated rheumatic disease characterized by fibrosis and vasculopathy of the skin and internal organs [256, 257]. It seriously affects the quality of life and mental health of patients [258, 259]. There is increasing evidence that the gut microbiota plays an important role in the pathogenesis of systemic sclerosis [260]. Volkmann et al. found that, compared with age- and sex-matched healthy controls, patients with systemic sclerosis had a decrease in beneficial commensal genera such as probiotics and *Clostridium* in the gut flora, while an increase in potentially pathogenic genera, including *Fusarium, Ruminococcus*, and rare γ-Proteobacteria [261]. Volkmann et al. found that patients with systemic sclerosis had less commensal bacteria and increased pathogenic bacteria than healthy people [262]. In addition, a large observational cohort study in Sweden found that patients with systemic sclerosis showed a reduction in brucella and/or clostridia [263]. Further studies found that while *Bifidobacteria* and *Lactobacilli* are normally reduced under inflammatory conditions, they are substantially increased in patients with systemic sclerosis [264]. In addition, dysbiosis of gut flora in patients with systemic sclerosis may directly contribute to the development of fibrosis in skin and internal organs. Mehta et al. demonstrated that early antibiotic exposure leads to persistent gut dysbiosis, which exacerbates skin and lung fibrosis later in the disease [265]. In addition, other fibrosis is also associated with gut microbiota, for example bacterial translocation is associated with liver fibrosis. Mazagova et al. treated conventional and sterile C57BL/6 mice with thioacetamide by gavage or intraperitoneal injection of carbon tetrachloride to induce liver injury, and found that the commensal flora had a protective effect on liver fibrosis in the model mice [266].

This meta-analysis showed that total GIT and HAQ-DI were not significantly improved by systemic sclerosis. However, the systematic review showed that probiotics improved patients with gastrointestinal symptoms such as diarrhea, abdominal pain, and gas/bloating/bloating [83]. However, due to the small number of RCTs, no firm conclusions can be drawn, and more RCTs are needed to confirm or revise the results.

### Gut microbiota-based therapies for T1DM

T1DM is an autoimmune disease characterized by the progressive destruction of insulin-secreting pancreatic β cells in pancreatic islets and is caused by a complex interaction between genetics and the environment [267, 268]. At present, it has been clear that the occurrence of T1DM is mainly mediated by immunity, and a variety of immune cells and their cytokines are involved in the destruction of pancreatic β cells [269]. The latest research found that both genetic and environmental factors play a role in the occurrence of T1DM, especially the intestinal flora affects the development of T1DM [270]. Animal experiments have shown that Th1/Th2 imbalance plays a key role in the occurrence and development of T1DM. Cytokines secreted by Th1 cell subsets, such as IL-2, IFN-γ, TNF-α, IL-12, and GM-CSF, can enhance the inflammatory response, mediate islet cell damage, and lead to the occurrence of T1DM. The cytokines secreted by Th2 cell subsets, such as IL-4 and IL-10, can inhibit the inflammatory response and play a certain role in alleviating the development of T1DM [271]. In addition, some studies have found that the proportion of CD4+ and CD8+ cells that can secrete IL-17 in the peripheral blood of T1DM patients is increased, and the number of CD4+ CD25+ Treg cells is significantly lower than that of the control group [272]. At present, many experiments have proved that probiotics can prevent the occurrence of T1DM by regulating immune cells and their cytokines, inhibiting inflammatory responses, and improving antibiotic sensitivity. *Lactobacillus casei* YIT 9018 supplementation can significantly reduce spleen CD8+ T cells and systemic inflammatory markers, indicating that probiotics may prevent the development of T1DM by reducing inflammatory response and blood sugar levels. In addition, *Lactobacillus equirum* M and *Lactobacillus kefir* K were screened in one study for their ability to promote glucagon-like peptide-1 (GLP-1) secretion from STC-1 cells. Two strains of lactic acid bacteria were fed to mice with streptozotocin-induced T1DM and found improvement in diabetes-related symptoms. The possible mechanisms include probiotics stimulating the secretion of GLP-1, inhibiting the production of pro-inflammatory factors and inflammatory cytokines, increasing the production of IL-10, and changing the intestinal flora [273]. The progression of T1DM was effectively alleviated in NOD mice after oral administration of *Lactobacillus*. The mechanism may be that probiotics inhibit the expression of IL-1β, reduce the release of indoleamine 2,3-dioxygenase, and promote the differentiation of intestinal CD103+ tolerogenic dendritic cells [274].

This meta-analysis shows that the addition of probiotics can improve blood glucose (lower HbA1c) in patients with T1DM and is relatively safe. However, due to the small number of RCTs, no firm conclusions can be drawn, and more RCTs are needed to confirm or revise the results.

### Gut microbiota-based therapies for OLP

OLP is a chronic oral mucosal epithelioid inflammatory disease that often occurs in middle-aged people over the age of 40 [275]. The etiological mechanism of OLP is still unclear, and it may be related to mental factors (such as fatigue, anxiety, stress), immune factors, endocrine factors, infectious factors, microcirculation factors, microbial imbalances, and certain systemic diseases (diabetes, infection, hypertension, digestive tract dysfunction) [276, 277]. It is generally believed that OLP is due to the mutual assistance of multiple cells, proteins in the cell matrix, and related chemokines to activate different pathways [278–280]. Recent studies have shown that OLP is associated with an imbalance in the human microbiota [281], which opens up new therapeutic prospects for its new intervention (probiotics). Regarding gut microbiota dysbiosis, Deng et al. analyzed the oral microbiota of OLP patients and found reductions in *Derxia*, *Haemophilus*, and *Pseudomonas* [282]. They demonstrated a positively correlated increase in TLR4 and NF-kB p65 in tissues and showed that shifts in the microbiota can contribute to the triggering of the inflammatory state that underlies disease onset and progression [282]. A significant reduction in the relative amount of *S. salivarius* was detectable in OLP patients [283]. Multiple studies have demonstrated dysbiosis of oral microbial communities in OLP patients, based on studies of host factors that make up the oral environment. Microbial communities in OLP trigger intracellular signaling pathways involved in oral pathology, which in turn lead to OLP pathologies such as keratinization, inflammation, and T cell responses [284–286].

However, this meta-analysis did not find an improvement in OLP with probiotics, which may be due to the small sample size, few RCTs included, and unstable study results. Therefore, more RCTs are needed in the future.

### Gut microbiota-based therapies for Crohn’s disease

Crohn’s disease is a chronic inflammatory disease of the gastrointestinal tract, characterized by periodic remission and relapse, involving the entire gastrointestinal tract, most often the terminal ileum and adjacent colon [287, 288]. The incidence of Crohn’s disease is high in Europe and North America, about 10–30/100,000 people [289, 290]. With the progress of industrialization, the incidence of Crohn’s disease in Asian populations continues to rise, especially in economically developed regions [291]. The chronic progression of inflammatory response in Crohn’s disease increases the risk of disability and seriously affects the quality of life of patients. The disease burden of patients with Crohn’s disease is heavy [292, 293]. The mainstream view holds that the “golden triangle” represented by intestinal epithelial cells (IEC), secretory IgA, and intestinal flora is one of the main factors leading to the pathogenesis of Crohn’s disease [294–296]. Studies have found that the types and numbers of bacteria in the gut of patients with Crohn’s disease are significantly different from those of normal people. The main manifestations are the decrease of *bifidobacteria, lactobacilli*, and *clostridium prazines*, and the increase of bacteria with strong mucoadhesion [297]. Previous studies have found that compared with healthy controls, patients with Crohn’s disease have significant intestinal flora imbalance, and the diversity and richness of the flora are reduced [298–300]. The gut microbiota of first-degree relatives of patients with Crohn’s disease exhibits a Crohn’s disease-like dysregulation pattern [301]. In addition, intestinal flora imbalance is associated with Crohn’s disease activity and disease progression [302–304], and intestinal flora is a key factor in postoperative recurrence of Crohn’s disease [305, 306], but there are also views that intestinal flora alterations in the composition and stability of the tract microbiota were not associated with either disease activity nor long-term disease course [307].

This systematic review showed that Crohn’s disease activity index, histological score, ESR, and CRP were significantly decreased after probiotic intervention, while hemoglobin was increased, and within the past 2 weeks, abdominal distension scores were significantly decreased and feeling good scores increased [91, 93]. Meanwhile, the median time to relapse was 16 ± 4 weeks in the probiotic group and 12 ± 4.3 weeks in the placebo group [92]. However, due to the small number of RCTs, no firm conclusions can be drawn, and more RCTs are needed to confirm or revise the results.

### Gut microbiota-based therapies for ulcerative colitis

Ulcerative colitis is a chronic nonspecific intestinal inflammatory disease of unknown etiology [308, 309]. Some patients can also cause various extraintestinal manifestations such as arthritis, eye diseases, and skin and mucous membrane lesions. A small number of severe patients may manifest as toxic megacolon, intestinal perforation, hemorrhage, and cancer, which endanger people’s lives [310, 311]. At present, it is believed that the pathogenesis of ulcerative colitis is related to various factors such as environmental factors, genetic factors, immune factors, and intestinal flora factors [312, 313]. It is generally believed that the imbalance of intestinal flora in patients is an important reason for the pathogenesis of ulcerative colitis [314]. Studies have found that the human gut microbiota plays an important role in the pathogenesis of ulcerative colitis and may determine the severity of intestinal inflammation. There is increasing evidence that the gut microbiota plays an important role in the pathogenesis of ulcerative colitis and may determine the severity of intestinal inflammation [296, 315]. Compared with healthy people, the changes of intestinal microorganisms in patients with ulcerative colitis were mainly reflected in the decrease of facultative anaerobic bacteria (such as *Clostridium species*, *Clostridium* IV cluster), and the increase of conditional pathogenic microorganisms, such as *Klebsiella*, *Enterobacter*, and *Proteus* [316]. In addition, studies have found that patients with ulcerative colitis have a reduced number of butyrate-producing bacteria in the gut microbiota in areas of active inflammation, such as *F. prausnitzii* and *Roseburia hominis* [317, 318]. In active ulcerative colitis patients, the concentration of butyrate in feces decreases, and the ability of intestinal mucosa to oxidize butyrate also decreases, but in patients with ulcerative colitis in remission, butyrate oxidation is at a normal level [317, 318]. In the clinical treatment of patients with ulcerative colitis, probiotics can restore the balance of intestinal flora and inhibit inflammatory response, and the side effects are smaller than traditional drug treatment [312, 313].

This meta-analysis also showed that probiotics can improve the endoscopic score of ulcerative colitis patients, improve the overall response rate (reduce inefficiency), reduce disease activity, and reduce CRP and ESR levels, and there are no obvious adverse events. However, due to the small number of RCTs, no firm conclusions can be drawn, and more RCTs are needed to confirm or revise the results.

## Conclusions

Gut microbiota-based therapies may have potential to treat celiac sprue, SLE and LN, JIA, psoriasis, fibromyalgia syndrome, PSS, MS, T1DM, Crohn’s disease and ulcerative colitis. However, while this therapy reduced pain in fibromyalgia syndrome, its effect on Fibromyalgia Impact Questionnaire scores was not significant. And for T1DM, this therapy may improve HbA1c, but its effect on total insulin requirements does not appear to be significant. Meanwhile, gut microbiota-based therapies may not improve the symptoms and/or inflammatory factor of spondyloarthritis and RA.

### Supplementary Information

**Additional file 1.** PRISMA 2020 Checklist. An checklist for reporting systematic reviews.

**Additional file 2.** Search Strategies for Pubmed and Embase.

**Additional file 3.** The characteristics of the included studies.

**Additional file 4.** The publication bias of endoscopy score in ulcerative colitis. The figure of publication bias of endoscopy score in ulcerative colitis.

**Additional file 5.** The publication bias of ineffective rate in ulcerative colitis. The figure of publication bias of ineffective rate in ulcerative colitis.

**Additional file 6.** The publication bias of disease activity in ulcerative colitis. The figure of publication bias of disease activity in ulcerative colitis.

**Additional file 7.** The publication bias of relapse rate in ulcerative colitis. The figure of publication bias of relapse rate in ulcerative colitis.

**Additional file 8.** The publication bias of adverse events in ulcerative colitis. The figure of publication bias of adverse events in ulcerative colitis.

**Additional file 9.** Sensitivity analysis of gut microbiota-based therapies for ulcerative colitis. A: Endoscopy Score; B: Ineffective rate; C: Disease activity.

## Data Availability

The data used to support the findings of this study are included within the article.

## Abbreviations

CI: Confidence interval
CIA: Collagen-induced arthritis
CNKI: China National Knowledge Infrastructure
DC: Dendritic cell
DMARD: Disease-modifying antirheumatic drug
JIA: Juvenile idiopathic arthritis
LN: Lupus nephritis
LPS: Lipopolysaccharide
MAPK: Mitogen-activated protein kinase
MS: Multiple sclerosis
OLP: Oral lichen planus
RA: Rheumatoid arthritis
RCT: Randomized controlled trial
RR: Relative risk
SAA: Serum amyloid A
SLE: Systemic lupus erythematosus
SMD: Standardized mean differences
T1DM: Type 1 diabetes mellitus
tTGA: Transglutaminase autoantibodies
VOC: Volatile organic compound
WMD: Weighted mean differences
