# Supplementary material for: Efficacy and safety of gut microbiota-based therapies in autoimmune and rheumatic diseases: a systematic review and meta-analysis of 80 randomized controlled trials
Source: BMC Med. 2024 Mar 13;22:110. doi: 10.1186/s12916-024-03303-4 (PMC10935932; doi:10.1186/s12916-024-03303-4)
Supplement: Supplementary file 2 — Additional file 2. Search Strategies for Pubmed and Embase. [file 12916_2024_3303_MOESM2_ESM.docx]

**Table S1.** Search Strategies for Pubmed and Embase

| **PubMed** | (probiotic OR synbiotic OR Lactobacillus OR Bifidobacterium OR Streptococcus thermophilus OR Lactococcus OR Bacillus subtilis OR Enterococcus OR Enterococcus faecium OR Enterococcus faecalis OR Saccharomyces OR Probiotics)  AND  (Autoimmune diseases OR Autoantibodies OR Autoimmunity OR Multiple Sclerosis OR Multiple Sclerosis, Chronic Progressive OR Multiple Sclerosis, Relapsing-Remitting OR Narcolepsy OR Hypersomnolence, Idiopathic OR Addison's Disease OR Diabetes Mellitus, Type 1 OR Graves Disease OR Hashimoto Disease OR Hepatitis, Autoimmune OR Coeliac Disease OR Crohn Disease OR Anemia, Pernicious OR Liver Cirrhosis, Biliary OR Colitis, Ulcerative OR Antiphospholipid Syndrome OR Purpura, Thrombocytopenic, Idiopathic OR Arthritis, Rheumatoid OR Mucocutaneous Lymph Node Syndrome OR Rheumatic Fever OR Giant Cell Arteritis OR Alopecia Areata OR Dermatitis Herpetiformis OR Vitiligo OR Scleroderma, Systemic OR Scleroderma, Localized OR Scleroderma, Diffuse OR Scleroderma, Limited OR Sjögren's syndrome OR Lupus Erythematosus, Systemic)  AND  (random* controlled trial [pt] OR controlled clinical trial* [pt] OR randomized [tiab] OR placebo [tiab] OR drug therapy [sh] OR random* [tiab] OR trial* [tiab] OR group* [tiab])  NOT  (animals [mh] NOT humans [mh]) |
| --- | --- |
| **EMBASE** | 1 Autoimmune diseases/  2 Autoantibodies/  3 Autoimmunity/  4 Multiple Sclerosis/  5 Multiple Sclerosis, Chronic Progressive/  6 Multiple Sclerosis, Relapsing-Remitting/  7 Narcolepsy/  8 Hypersomnolence, Idiopathic/  9 Addison's Disease/  10 Diabetes Mellitus, Type 1/  11 Graves Disease/  12 Hashimoto Disease/  13 Hepatitis, Autoimmune/  14 Coeliac Disease/  15 Crohn Disease/  16 Anemia, Pernicious/  17 Liver Cirrhosis, Biliary/  18 Colitis, Ulcerative/  19 Antiphospholipid Syndrome/  20 Purpura, Thrombocytopenic, Idiopathic/  21 Arthritis, Rheumatoid/  22 Mucocutaneous Lymph Node Syndrome/  23 Rheumatic Fever/  24 Giant Cell Arteritis/  25 Alopecia Areata/  26 Dermatitis Herpetiformis/  27 Vitiligo/  28 Scleroderma, Systemic/  29 Scleroderma, Localized/  30 Scleroderma, Diffuse/  31 Scleroderma, Limited/  32 Sjögren's syndrome/  33 Lupus Erythematosus, Systemic/  34 (autoimmune adj1 (disease$ or response$)).ti,ab,kw  35 (autoantibod$ or autoimmunit$).ti,ab,kw  36 ((multiple or disseminated) adj1 scleros$).ti,ab,kw  37 (MS or narcolepsy or iddm or sprue or ileocolitis or vitiligo or RA).ti,ab,kw  38 ((gelineau$ or narcoleptic) adj1 syndrome$).ti,ab,kw  39 (narcolepsy adj2 cataplexy adj1 syndrome$).ti,ab,kw  40 (paroxysmal adj1 sleep$).ti,ab,kw  41 (addison$ adj1 disease$).ti,ab,kw  42 (primary adj1 (adrenal or adrenocortical) adj1 insufficienc$).ti,ab,kw  43 (primary adj1 hypoadrenalism$).ti,ab,kw  44 (diabetes adj1 mellitus).ti,ab,kw  45 (type adj1 ("1" or one) adj1 diabete$).ti,ab,kw  46 (autoimmune adj2 (diabete$ or hyperthyroidism or hepatitis or hepatitides)).ti,ab,kw  47 ((grave$ or basedow$) adj1 disease$).ti,ab,kw  48 (exophthalmic adj1 goiter$).ti,ab,kw  49 (hashimoto$ adj2 (disease$ or struma or syndrome$ or thyroiditides or thyroiditis)).ti,ab,kw  50 (chronic adj1 lymphocytic adj1 (thyroiditides or thyroiditis)).ti,ab,kw  51 (coeliac adj1 (disease$ or sprue)).ti,ab,kw  52 (gluten adj2 enteropath$).ti,ab,kw  53 (nontropical adj1 sprue).ti,ab,kw  54 (crohn$ adj1 (disease$ or enteritis)).ti,ab,kw  55 (granulomatous adj1 (colitis or enteritis)).ti,ab,kw  56 ((regional or terminal) adj1 (enteritis or ileitides or ileitis)).ti,ab,kw  57 (inflammatory adj1 bowel adj1 disease$).ti,ab,kw  58 ((pernicious or addison$) adj1 (anaemia or anemia)).ti,ab,kw  59 ((biliary or liver) adj1 (cirrhosis or cirrhoses)).ti,ab,kw  60 (ulcerative adj1 colitis).ti,ab,kw  61 (colitis adj1 gravis).ti,ab,kw  62 (idiopathic adj1 proctocolitis).ti,ab,kw  63 (antiphospholipid adj3 syndrome$).ti,ab,kw  64 (hughes adj1 syndrome).ti,ab,kw  65 ((immune or autoimmune or idiopathic) adj1 thrombocytopen$).ti,ab,kw  66 (werlhof$ adj1 disease$).ti,ab,kw  67 ((rheumatoid or rheumatic) adj1 arthritis).ti,ab,kw  68 (kawasaki adj1 (disease$ or syndrome$)).ti,ab,kw  69 (rheumatic adj1 (fever$ or arthritides)).ti,ab,kw  70 ((articular or inflammatory or polyarthritis) adj1 (rheumatism$ or rheumatica$)).ti,ab,kw  71 ((temporal or cranial) adj1 (arteritis or arteritides)).ti,ab,kw  72 (giant adj1 cell adj2 (arteritis or aortitides or aortitis)).ti,ab,kw  73 (horton$ adj3 (disease$ or arteritis)).ti,ab,kw  74 (alopecia adj1 (areata or circumscripta)).ti,ab,kw  75 (dermatitis adj1 herpetiformis).ti,ab,kw  76 (duhring$ adj1 disease$).ti,ab,kw  77 (scleroderma$ or dermatosclerosis or morphea$).ti,ab,kw  78 (systemic adj1 sclerosis).ti,ab,kw  79 ((Sjögren$ or sicca) adj1 syndrome).ti,ab,kw  80 (lupus adj1 erythematosus).ti,ab,kw  81 (libman adj1 sacks adj1 disease).ti,ab,kw  82 (mucocutaneous adj1 lymph adj1 node adj1 syndrome).ti,ab,kw  83 or/1-82  84 probiotic*.tw.  85 synbiotic*.tw.  86 'Lactobacillus'/exp  87 lactobacill*.tw.  88 bacill*.tw.  89 'Bifidobacterium'/exp  90 (bifidus or bifidobacter*).tw.  91 'Streptococcus thermophilus'/exp  92 streptococcus thermophilus.tw.  93 streptococc*.tw.  93 'Lactococcus'/exp  94 lactococc*.tw.  95 'Bacillus subtilis'/  96 bacillus subtilis.tw.  97 'Enterococcus'/exp  98 'Enterococcus faecium'/exp or 'Enterococcus faecalis'/  99 'Saccharomyces'/exp  100 saccharomyc*.tw.  101 leuconostoc.tw.  102 pediococc*.tw.  103 bulgarian bacillus.tw.  104 (beneficial adj3 bacter*).tw.  105 (Escherichia coli or "E. coli").tw.  106 Yeast.tw.  107 (fungus or fungi).tw.  108 (VSL# 3 or VSL 3).tw.  109 'Probiotics'/exp  110 or/84-109  111 random$.tw.  112 factorial$.tw.  113 (crossover$ or cross over$ or cross-over$).tw.  114 placebo$.tw.  115 single blind.mp.  116 double blind.mp.  117 triple blind.mp.  118 (singl$ adj blind$).tw.  119 (double$ adj blind$).tw.  120 (tripl$ adj blind$).tw.  121 assign$.tw.  122 allocat$.tw.  123 crossover procedure/  124 double blind procedure/  125 single blind procedure/  126 triple blind procedure/  127 randomized controlled trial/  128 or/111-127  129 83 and 110  130 128 and 129 |
