# Supplementary figures and images for: Efficacy and safety of gut microbiota-based therapies in autoimmune and rheumatic diseases: a systematic review and meta-analysis of 80 randomized controlled trials
Source: BMC Med. 2024 Mar 13;22:110. doi: 10.1186/s12916-024-03303-4 (PMC10935932; doi:10.1186/s12916-024-03303-4)

SND of effect estimate

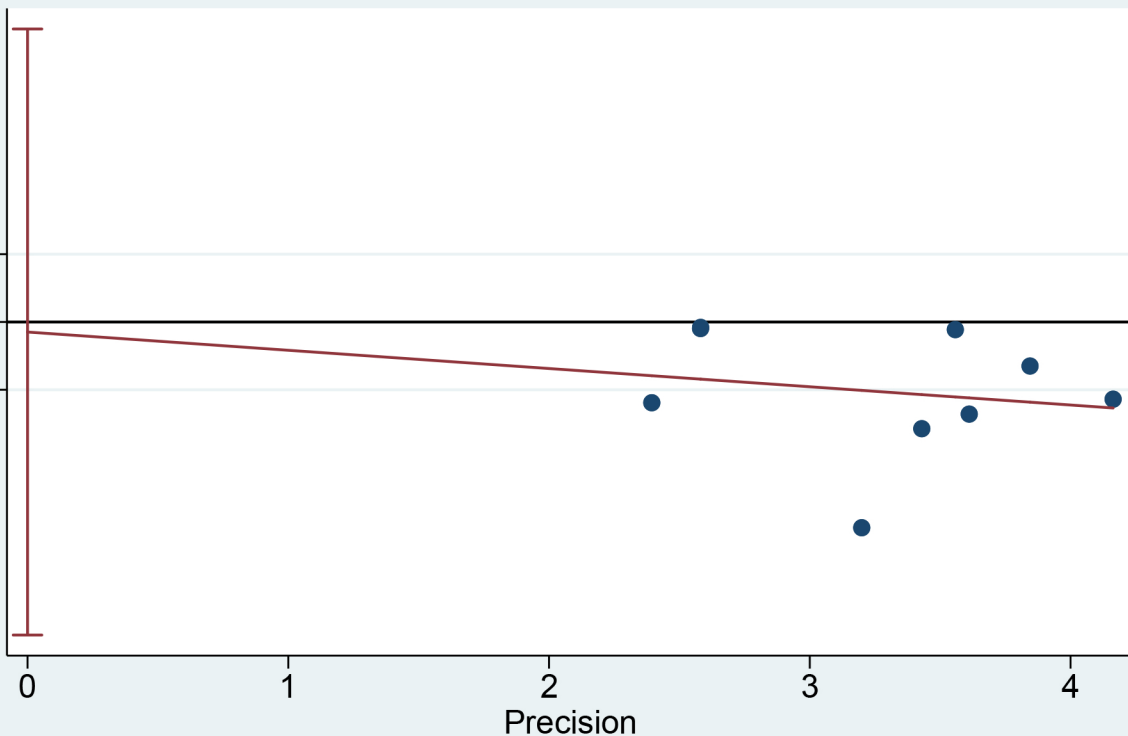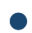

Study

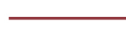

regression line

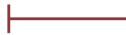

95% CI for intercept

Supplement: Supplementary file 4 — Additional file 4. The publication bias of endoscopy score in ulcerative colitis. The figure of publication bias of endoscopy score in ulcerative colitis. [file 12916_2024_3303_MOESM4_ESM.pdf]

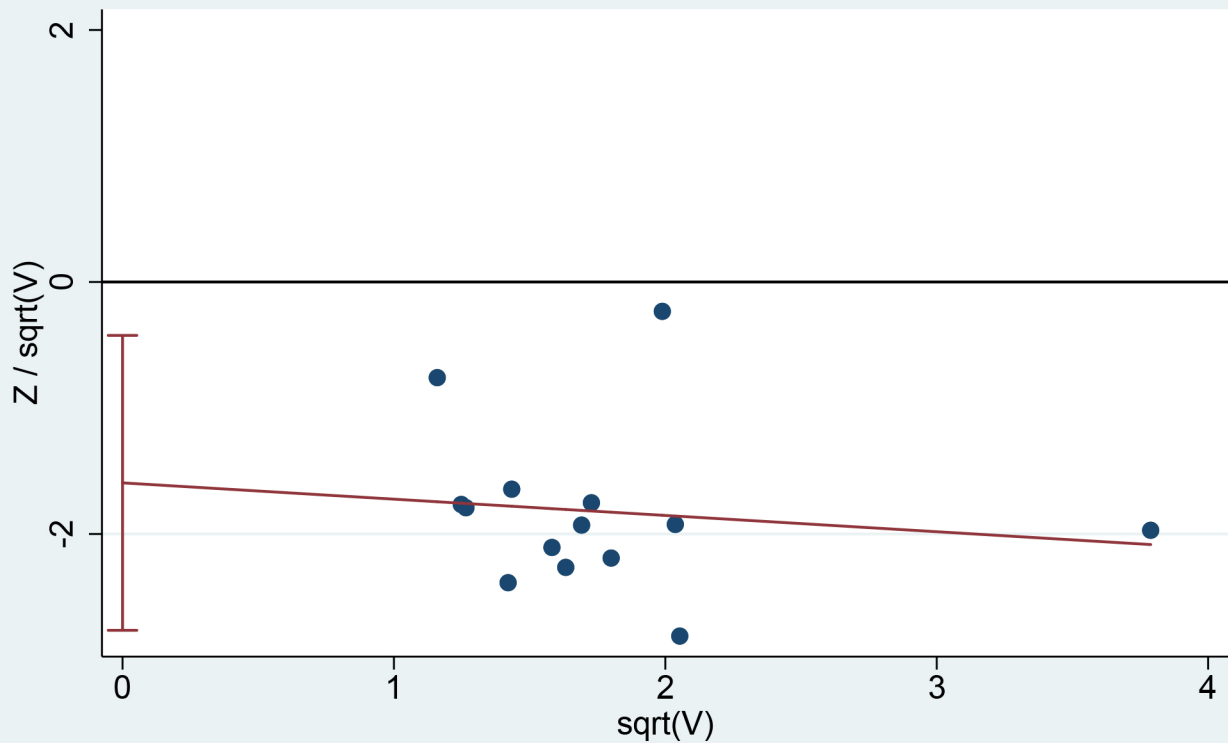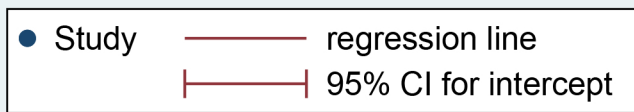

Supplement: Supplementary file 5 — Additional file 5. The publication bias of ineffective rate in ulcerative colitis. The figure of publication bias of ineffective rate in ulcerative colitis. [file 12916_2024_3303_MOESM5_ESM.pdf]

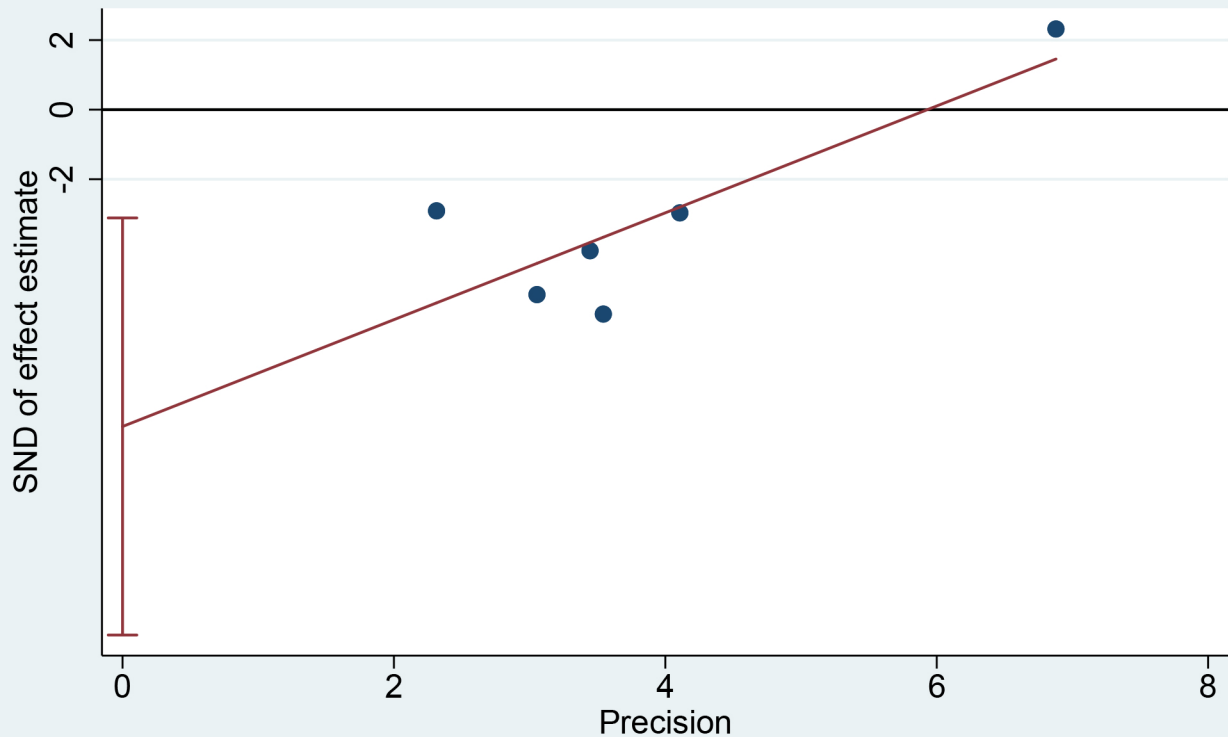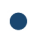

Study

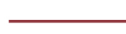

regression line

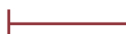

95% CI for intercept

Supplement: Supplementary file 6 — Additional file 6. The publication bias of disease activity in ulcerative colitis. The figure of publication bias of disease activity in ulcerative colitis. [file 12916_2024_3303_MOESM6_ESM.pdf]

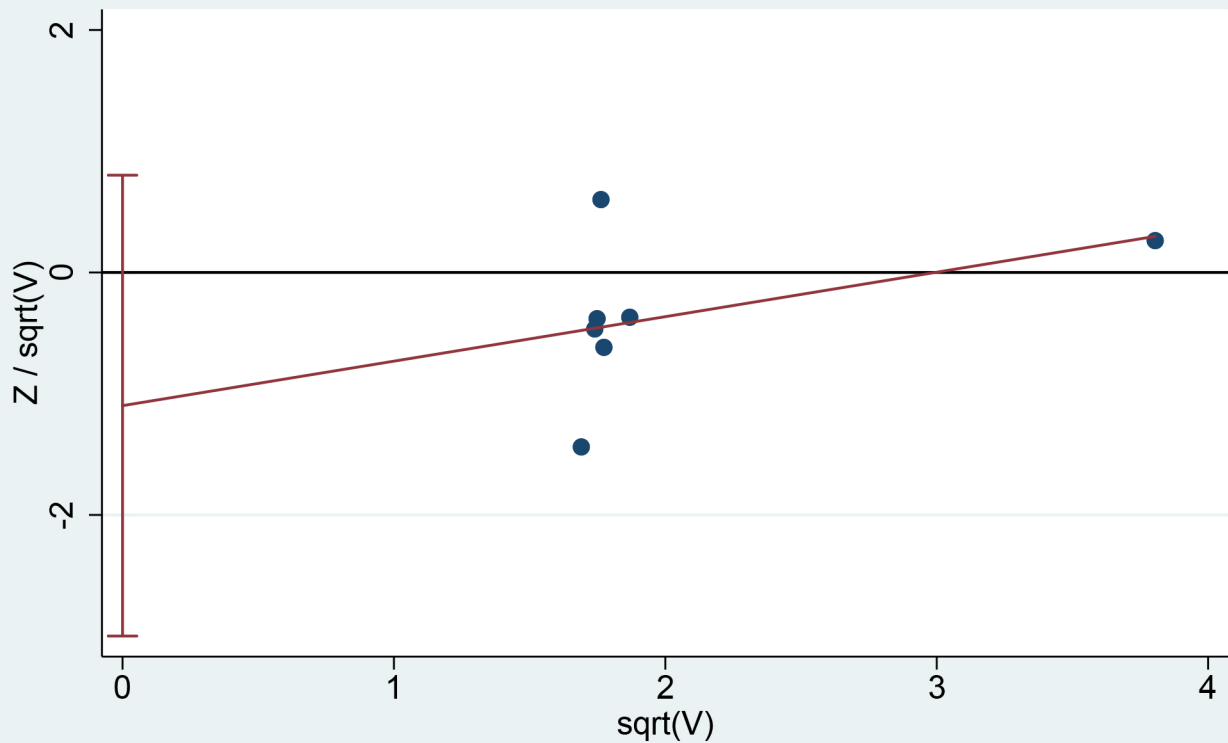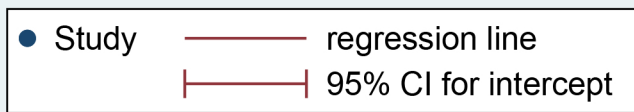

Supplement: Supplementary file 7 — Additional file 7. The publication bias of relapse rate in ulcerative colitis. The figure of publication bias of relapse rate in ulcerative colitis. [file 12916_2024_3303_MOESM7_ESM.pdf]

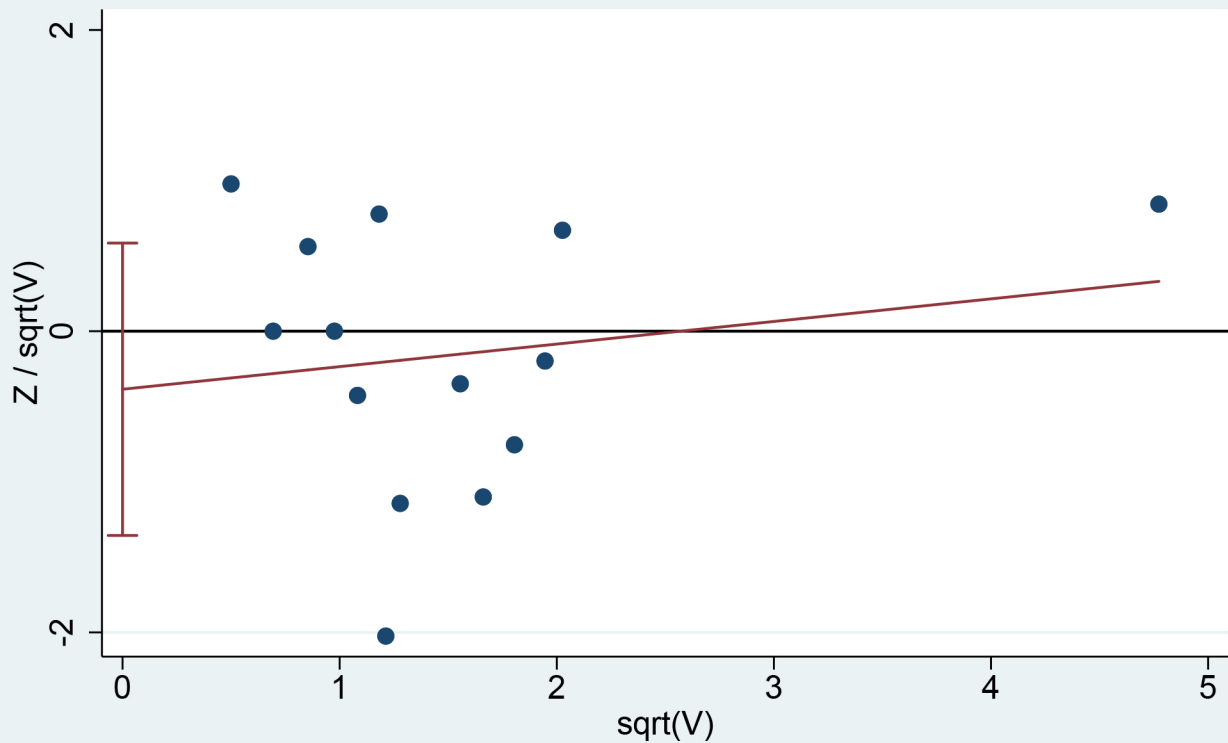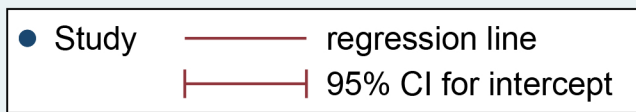

Supplement: Supplementary file 8 — Additional file 8. The publication bias of adverse events in ulcerative colitis. The figure of publication bias of adverse events in ulcerative colitis. [file 12916_2024_3303_MOESM8_ESM.pdf]

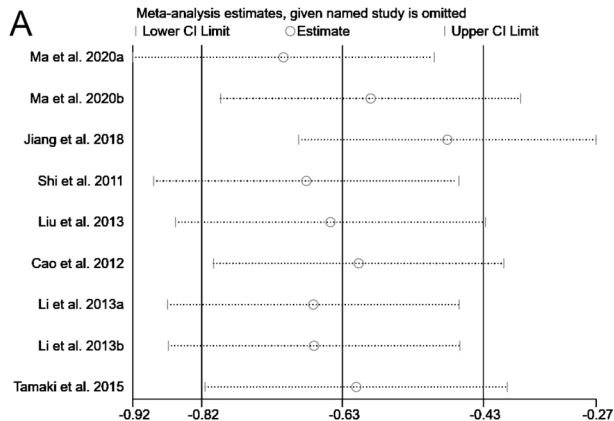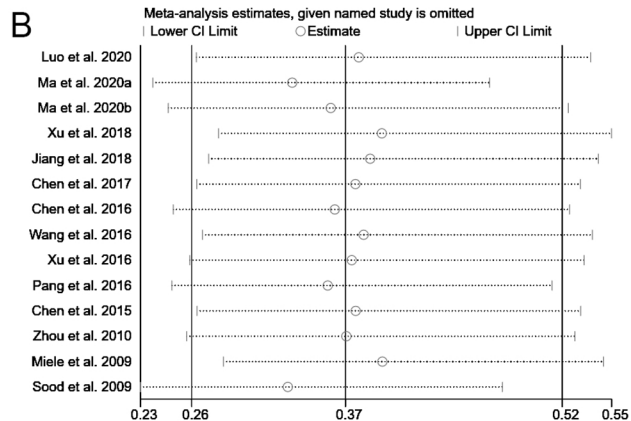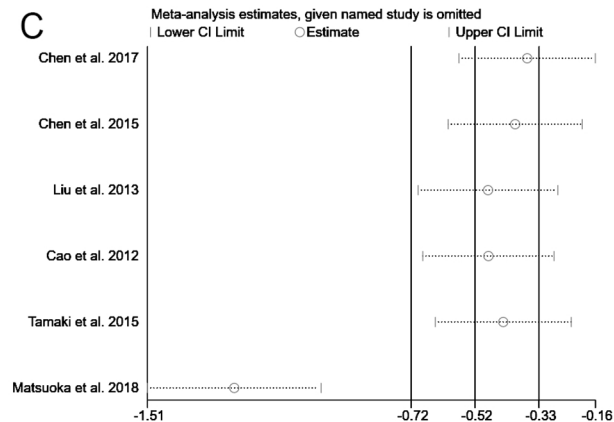

Supplement: Supplementary file 9 — Additional file 9. Sensitivity analysis of gut microbiota-based therapies for ulcerative colitis. A: Endoscopy Score; B: Ineffective rate; C: Disease activity. [file 12916_2024_3303_MOESM9_ESM.pdf]
